# Synthetic tools for studying the chemical biology of InsP_8_ †
†Electronic supplementary information (ESI) available: Data deposition: atomic coordinates and structure factors have been deposited in the Protein Data Bank, www.pdb.org (PDB ID codes 5BYA and 5BYB). See DOI: 10.1039/c5cc05017k



**DOI:** 10.1039/c5cc05017k

**Published:** 2015-08-14

**Authors:** Andrew M. Riley, Huanchen Wang, Stephen B. Shears, Barry V. L. Potter

**Affiliations:** a Wolfson Laboratory of Medicinal Chemistry , Department of Pharmacy and Pharmacology , University of Bath , Claverton Down , Bath , BA2 7AY , UK; b Inositol Signaling Group , Laboratory of Signal Transduction , National Institute of Environmental Health Sciences , National Institutes of Health , Research Triangle Park , North Carolina , USA; c Department of Pharmacology , University of Oxford , Mansfield Road , Oxford , OX1 3QT , UK . Email: barry.potter@pharm.ox.ac.uk ; Fax: +44-1865-271853 ; Tel: +44-1865-271945

## Abstract

To synthesise stabilised mimics of InsP_8_, the most phosphorylated inositol phosphate signalling molecule in Nature, we replaced its two diphosphate (PP) groups with either phosphonoacetate (PA) or methylenebisphosphonate (PCP) groups. Utility of the PA and PCP analogues was verified by structural and biochemical analyses of their interactions with enzymes of InsP_8_ metabolism.

## Abstract 2

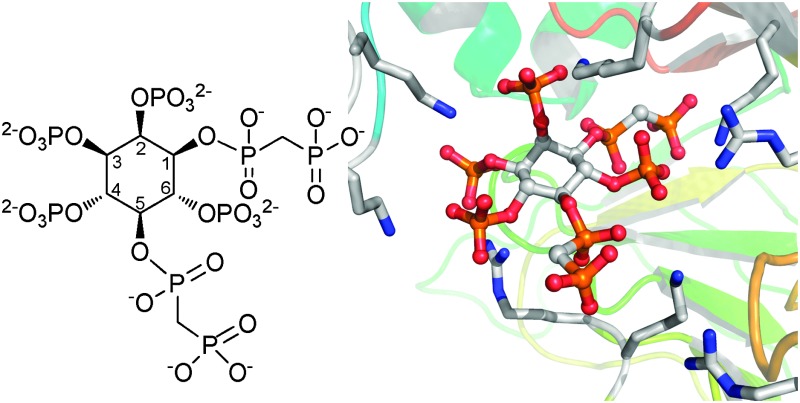
Synthetic mimics of InsP_8_, Nature's most phosphorylated inositol phosphate, interact differentially with the kinase PPIP5K2 and provide stabilised, complementary tools to investigate this orphan signal.

The *myo*-inositol phosphates (InsPs) are a family of intracellular signalling molecules containing combinatorial arrangements of monophosphate (P) and diphosphate (PP) groups arranged around the hexahydroxycyclohexane ring of *myo*-inositol (Ins).^1^ There is much current interest in the specialised chemistry and biology of the diphosphoinositol polyphosphates (PP-InsPs, inositol pyrophosphates);^2^ recent research has highlighted the central roles that PP-InsPs play in cellular and organismic homeostasis in all eukaryotes. For example, PP-InsPs regulate DNA repair,^3^ immunity^4,5^ and metabolic homeostasis.^6–8^

The most studied of the PP-InsPs are 5-InsP_7_, 1-InsP_7_ and InsP_8_ (Fig. 1), which are formed from InsP_6_ by InsP_6_ kinases (IP6Ks) and diphosphoinositol pentakisphosphate kinases (PPIP5Ks).^9^ The diphosphate groups that are produced by IP6K and PPIP5K are hydrolysed by a family of PP-InsP diphosphohydrolases (DIPPs), leaving monophosphate groups and liberating inorganic orthophosphate (Pi).^10^

**Fig. 1:**
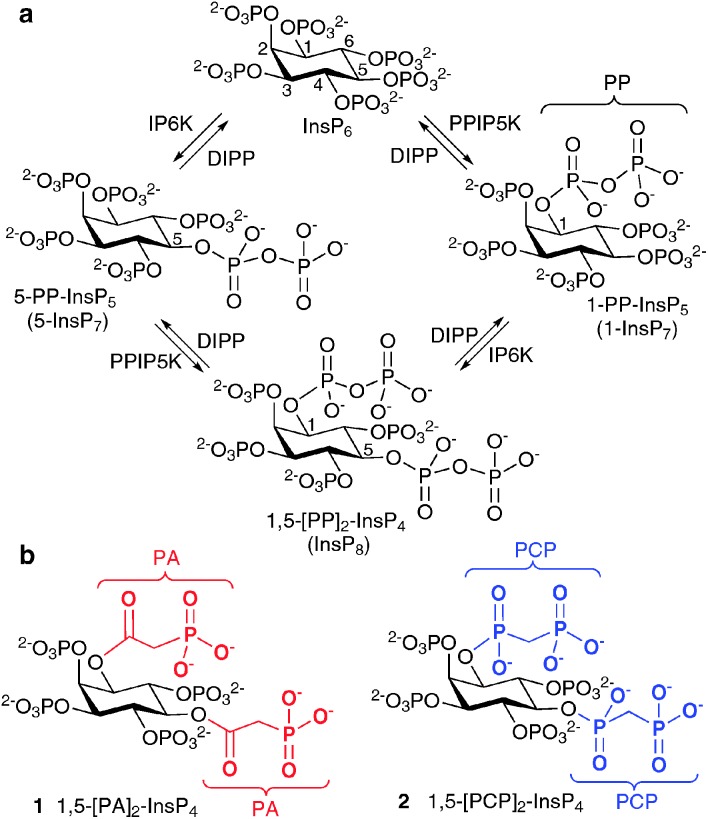
(a) Biosynthesis of diphosphoinositol polyphosphates from *myo*-inositol hexakisphosphate (InsP_6_). IP6K, inositol hexakisphosphate 5-kinase; PPIP5K, diphosphoinositol pentakisphosphate kinase; DIPP, diphosphoinositol polyphosphate phosphohydrolase; (b) structures of synthetic α-phosphonoacetic acid ester (PA) analogue **1** and methylenebisphosphonate (PCP) analogue **2**.

Evidence has been obtained that the PP-InsPs non-enzymatically pyrophosphorylate a range of target proteins.^11^ It has also been reported that 5-InsP_7_ produces a separate range of cellular effects by interacting with pleckstrin homology (PH) domains of proteins.^12^ Additionally, in response to viral invasion, 1-InsP_7_ was recently shown to stimulate phosphorylation of IRF3, an activator of interferon transcription.^5^ No independent function for InsP_8_ has, however, yet been shown. Nevertheless, in mammalian cells, levels of InsP_8_ are regulated in a stimulus-dependent fashion, increasing several-fold following osmotic stress or thermal challenge,^13^ whereas bioenergetic stress decreases InsP_8_ concentrations.^7^ Indeed, InsP_8_ is the only PP-InsP so far shown to exhibit such acute, stimulus-dependent changes in its levels. Such responses are typically hallmarks of a signalling event, in which the concentration-dependent influence of a messenger upon a target protein (a receptor) is transduced into a biological response. It is therefore reasonable to consider the existence of InsP_8_ “receptors”.

The structure of mammalian InsP_8_ has been identified^14,15^ as 1,5-[PP]_2_-InsP_4_ (Fig. 1) and a chemical synthesis^16^ has provided isomerically pure material in the amounts required for detailed biological studies with cell extracts. However, in such experiments, the PP-InsPs are rapidly metabolised by phosphatases. Therefore, there is a need for synthetic InsP_8_-based probes, especially stabilised analogues in which the labile diphosphate (PP) is replaced with mimics more resistant to chemical and enzymatic degradation. Such compounds could be used to screen for InsP_8_ receptors in cellular or tissue lysates and may have greater potential for further synthetic elaboration than InsP_8_ itself. They may also be useful as mechanistic probes because they cannot substitute for the ability of InsP_8_ to transfer a phosphate group to target proteins.^11^

Previously, we reported the syntheses of analogues of 5-InsP_7_, in which the PP group is replaced with an α-phosphonoacetic acid (PA) ester.^17^ Others have developed methylenebisphosphonate (PCP) analogues of 1- and 5-InsP_7_.^18^ The synthesis of the corresponding InsP_8_ mimics is considerably more challenging, requiring introduction of two PP surrogates around an asymmetrically substituted *myo*-inositol hub of known absolute configuration. Here we report the syntheses of the PA and PCP analogues of InsP_8_, namely, 1,5-[PA]_2_-InsP_4_ (**1**) and 1,5-[PCP]_2_-InsP_4_ (**2**), respectively.

The syntheses of **1** and **2** (Scheme 1) begins with diol (–)-**3**, obtained by regioselective reduction of 2,4,6-tri-*O*-benzyl *myo*-inositol orthobenzoate,^19^ followed by optical resolution of racemic **3** *via* the formation of diastereoisomeric monocamphanate esters^20^ (see ESI† for details).‡
‡The required starting material (–)-**3** was found to have the opposite specific rotation to that previously reported for 1d-2,3,4,6-tetra-*O*-benzyl-inositol.^20^ The correct absolute configuration for (–)-**3** was determined in the present work (see ESI† for details). For the synthesis of 1,5-[PA]_2_-InsP_4_ (**1**), carbodiimide-mediated esterification of the two free hydroxyl groups in (–)-**3** with diethylphosphonoacetic acid (**4**) proceeded smoothly to give diester **5**. Hydrogenolytic cleavage of the benzyl protecting groups allowed isolation of tetraol **6**, while avoiding the facile ester 1,2-migration. Phosphitylation of the exposed hydroxyl groups at C-2, C-3, C-4 and C-6 using bis(benzyloxy)-diisopropylaminophosphine, followed by oxidation gave **7**. Treatment with TMSBr removed benzyl and ethyl ester protecting groups on phosphates and phosphonates respectively to give the per-silylated intermediate. Finally, cleavage of silyl esters with methanol followed by a simple work-up with aqueous triethylammonium bicarbonate gave 1,5-[PA]_2_-InsP_4_ (**1**) as the triethylammonium salt.§
§Although the acyl esters in **1** will be susceptible to hydrolytic cleavage at high pH, the ^31^P NMR spectrum of a solution of the triethylammonium salt of **1** in D_2_O was unchanged after >1 year at 4 °C.


**Scheme 1:**
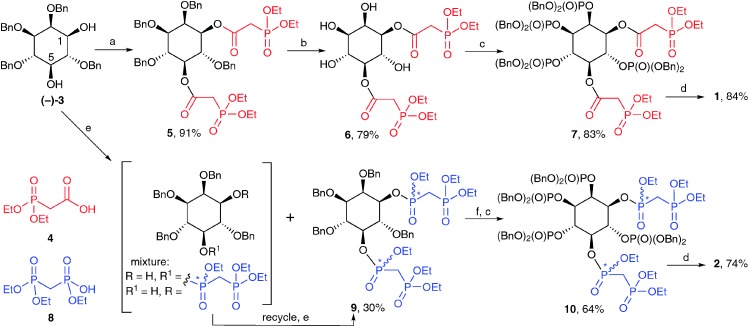
Synthesis of **1** and **2**. *Reagents and conditions*: (a) EDAC, **4**, CH_2_Cl_2_; (b) H_2_, Pd(OH)_2_/C, MeOH, H_2_O; (c) i. (BnO)_2_PNPri2, 5-phenyl-1*H*-tetrazole, CH_2_Cl_2_; ii. *m*CPBA, CH_2_Cl_2_; (d) i. TMSBr, CH_2_Cl_2_; ii. MeOH, TEAB; (e) (EtO)_2_P(O)CH_2_P(O)(OEt)Cl, DIPEA, CH_2_Cl_2_; (f) H_2_, Pd(OH)_2_/C, MeOH, THF, H_2_O, AcOH. TEAB, aqueous triethylammonium bicarbonate; Bn, benzyl. Yields are shown in respect of each step. Stereogenic phosphorus atoms are indicated by an asterisk.

The synthesis of 1,5-[PCP]_2_-InsP_4_ (**2**) from (–)-**3** was more challenging than the analogous synthesis of **1**, essentially due to two complicating factors. First, introduction of two tri-protected PCP moieties at O-1 and O-5 of (–)-**3** results in the formation of stereogenic centres at P-1 and P-5. The required product will therefore consist of a mixture of four diastereoisomers.

Second, phosphonylation of the sterically hindered secondary hydroxyl groups in (–)-**3** proved to be much more difficult than their acylation. Initial attempts at carbodiimide-mediated condensation of (–)-**3** with the triethyl ester of methylenebisphosphonic acid (**8**) gave no product. Reaction of (–)-**3** with the phosphonochloridate derived from **8** in pyridine gave only the 1-bisphosphonate as a mixture of two diastereoisomers. Trials using this phosphonochloridate with other bases and solvents gave the desired product **9**, but only in unacceptably low yields. More promising results were obtained using DIPEA in dichloromethane;¶
¶We found that the nature and pattern of protecting groups strongly influenced the outcome of the reaction. Phosphonylation of less sterically hindered substrates at either O-1 or O-5 was high-yielding under these conditions. under these conditions, **9** was obtained in 15% yield after flash chromatography, together with larger amounts of monophosphonylated material (in this case, a mixture of 1- and 5-bisphosphonates), which could be recycled to give more **9**. In this way, **9** was obtained as an inseparable mixture of four diastereoisomers (ratio approx. 3 : 3 : 1 : 1 by NMR) in a total yield of 30% after one round of recycling. Further recycling to give more **9** was possible, but was not carried out.

Removal of the benzyl protecting groups from **9** followed by phosphitylation and oxidation gave fully protected **10**, again as a mixture of four diastereoisomers. The NMR spectra of **10** were highly complex, with at least 30 lines in the ^31^P NMR spectrum (see ESI†). However, on global deprotection with TMSBr followed by cleavage of silyl esters with methanol, the stereogenic centres at phosphorus were abolished, at last revealing the expected pattern of NMR signals for 1,5-[PCP]_2_-InsP_4_ (**2**), which was isolated as the triethylammonium salt after ion-exchange chromatography.

When we incubated either InsP_8_ analogue **1** or **2** with DIPP, we did not detect any Pi release. Next, we examined the interactions of **1** and **2** with the highly specific kinase domain of human PPIP5K2 (PPIP5K2^KD^). We have previously found that the reaction catalysed by PPIP5K2^KD^ is reversible *in vitro*, yielding ATP when PPIP5K2^KD^ is incubated with ADP and InsP_8_.^21^ Detection of the generated ATP with luciferin/luciferase provides a sensitive assay for the reverse kinase reaction.^17,21,22^ No ATP was detected using **1** or **2** in these assays (data not shown).

Previously,^15^ we obtained enzyme-product complexes by soaking InsP_8_ into crystals of PPIP5K2^KD^ containing ADP. In the present work, we soaked product analogues **1** and **2** into similarly prepared crystals of PPIP5K2^KD^. X-ray analyses showed that both compounds bound to the catalytic site of PPIP5K2^KD^ (Fig. 2). Significantly, the PA-containing analogue **1** was also observed in a second binding site, the function of which was previously demonstrated to enhance capture of substrate from the bulk phase.^22^ In this respect, compound **2** more closely mimics natural InsP_8_, which was also found to occupy exclusively the catalytic site.^15,16^ This should not be taken to mean that InsP_8_ and **2** do not also bind to the capture site but that, presumably, they occupy it only transiently. Thus, the PA compound **1** provides additional insights into a subsequent step of the catalytic cycle; the transfer of newly-formed InsP_8_ to the capture site prior to release. Notably, the 1-PA group of **1** is solvent-exposed and has no interactions with the capture/release site. This is consistent with the requirement that the site must bind both substrate (without 1-PP) and product (with 1-PP); it also suggests a suitable attachment point for reporter groups in probes designed to target this site.

**Fig. 2:**
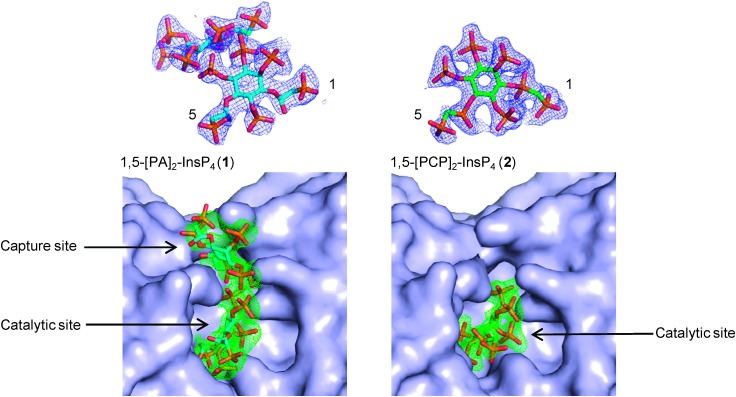
Refined 2*F*_o_–*F*_c_ maps contoured at 1.0 *σ* for both compounds (top) and simulated annealing omit maps (*F*_o_–*F*_c_, bottom panels) contoured at 2.0 *σ* for 1,5-[PA]_2_-InsP_4_ (**1**) and 3.0 for 1,5-[PCP]_2_-InsP_4_ (**2**). Compounds are shown as stick models. Carbon atoms are shown in cyan (**1)** or green (**2**), oxygen atoms in red, nitrogen atoms in blue, and phosphorus atoms in orange. The protein is shown as a surface representation.

In conclusion, we report the syntheses of two stabilised analogues of InsP_8_; 1,5-[PA]_2_-InsP_4_ (**1**) and 1,5-[PCP]_2_-InsP_4_ (**2**). Our observation that the PA analogue **1** occupies the capture/release site in PPIP5K2 reveals how InsP_8_ mimics can give insight into different enzyme states within the overall catalytic cycle. Stabilised mimics will also be useful for identifying receptors within cell lysates. Since these two new probes might interact with receptors in subtly different ways, the use of both may increase opportunities to capture InsP_8_ receptors. The differing and complementary interactions of **1** and **2** with PPIPK2 (Fig. 2) illustrate this point.

Cellular levels of InsP_8_ are subject to stimulus-dependent regulation, yet it remains an orphan signal; no specific InsP_8_ receptor has yet been identified. Nevertheless, InsP_8_, which possesses the most crowded array of phosphate groups in Nature, demands from the cell a significant investment in energy to sustain its levels against a backdrop of high ongoing turnover. With recent advances in delivering PP-InsPs (and hence PP-InsP mimics) into cells,^23^ it should now become possible to screen for biological effects of InsP_8_, and InsP_8_ mimics, in intact cells.

B.V.L.P. is a Wellcome Trust Senior Investigator (Grant 101010). This research was also supported by the Intramural Research Program of the NIH/National Institute of Environmental Health Sciences.
