# Supplementary material for: Synthetic tools for studying the chemical biology of InsP8
Source: Chem Commun (Camb). 2015 Aug 14;51(63):12605–8. doi: 10.1039/c5cc05017k (PMC4643724; doi:10.1039/c5cc05017k)
Supplement: Supplementary file 1 [file CC-051-C5CC05017K-s001.pdf]

Electronic Supplementary Material (ESI) for Chemical Communications.
This journal is © The Royal Society of Chemistry 2015

Supplementary Information

Synthetic tools for studying the chemical biology of $InsP_8$

Andrew M. Riley,[a] Huanchen Wang,[b] Stephen B. Shears[b] and Barry V. L. Potter*[a,c]

[a] Wolfson Laboratory of Medicinal Chemistry, Department of Pharmacy and Pharmacology, University of Bath, Claverton Down, Bath, BA2 7AY, UK.

[b] Inositol Signaling Group, Laboratory of Signal Transduction, National Institute of Environmental Health Sciences, National Institutes of Health, Research Triangle Park, North Carolina, USA.

[c] Department of Pharmacology, University of Oxford, Mansfield Road, Oxford, OX1 3QT, UK.

Table of Contents

Enzyme assays S2

Protein expression, purification, crystallisation and structure determination S2

Data collection and structure refinement statistics S3

General chemistry methods S4

Synthesis and determination of absolute configuration of 1D-2,3,4,6-tetra-*O*-benzyl-*myo*-inositol [(–)-3] S5–S10

Synthesis and characterisation of compounds 1, 2 and 5–10 S11–S17

NMR spectra S18–S35

References S36

DIPP1 phosphatase assay and reverse kinase assay

A colorimetric assay[1] was used to record the DIPP1-catalysed release of inorganic phosphate (Pi) from 50 μM compounds at 25 °C for 18 hours in 100 μL reaction mixtures containing 20mM HEPES, pH 7.2, 100 mM KCl, 0.6 mM $MgCl_2$ and 100μg/mL purified DIPP1.

A luminescence-based assay[2,3] was used to measure ATP accumulation on incubation of 100 μL reaction mixtures containing 10 μM compounds, 20 mM Tris-HCl, pH 7.5, 10 mM $MgCl_2$, 0.1 mM ADP and 2 μg/mL enzyme at 37 °C for 60 min.

Protein Expression, Purification, Crystallisation and Structure Determination

The kinase domain of human PPIP5K2 ($PPIP5K2^{KD}$; residues 41-366) was sub-cloned, expressed and purified as before.[4] The catalytic domain $PPIP5K2^{KD}$ was crystallised by hanging drop vapour diffusion against a well buffer of 12% (w/v) PEG 3350, 20 mM $MgCl_2$, 0.1 M HEPES, pH 7.0, 2 mM $CdCl_2$, 1 mM ATP at 4 ˚C. The crystals were transferred to a stabilising buffer containing 22% (w/v) PEG 3350, 10 mM $MgCl_2$, 0.1 M sodium acetate, pH 5.2 at 4 ˚C and the crystals were then soaked under the above stabilising buffer for three days with 2 mM compounds **1** or **2,** respectively. Cryosolvent was prepared by adding 33% ethylene glycol into the soaking buffer. Diffraction data were collected using APS beamlines 22-BM. All data were processed with the program HKL2000. The structure was determined using rigid body and direct Fourier synthesis, and refined with the equivalent and expanded test sets. The structure was further manually rebuilt with COOT and refined with REFMAC from the CCP4 package. Ligand topology and parameter files were prepared using the PRODRG server. The molecular graphics representations were prepared with the program PyMol (Schrödinger, LLC). Atomic coordinates and structure factors have been deposited with the Protein Data Bank with accession codes: 5BYA and 5BYB.

Supplementary Table. Data collection and structure refinement statistics

**PDB Accession Code**	5BYB	5BYA
**Data collection**		
	1,5-PA-InsP_4 (**1**)	1,5-PCP-InsP_4 (**2**)
Cell dimensions (a,b, c (Å))	88.8 110.4 41.4	88.6 110.4 41.4
Resolution (Å)*	50-2.3 (2.35)	50-1.90(1.93)
Rsym *	0.114(0.438)	0.113(0.668)
I/σI*	12.8(3.3)	15.8(2.4)
Completeness (%)*	99.2(95.5)	99.9(99.9)
Redundancy *	4.7 (4.7)	6.6 (6.3)
**Refinement**		
Resolution(Å)*	35.57-2.30(2.35)	35.57-1.90(1.95)
No. reflections	17585	30891
R_{work} *	18.9(20.4)	14.5(17.9)
R_{free} *	24.6(33.5)	19.9(26.8)
**No. atoms**		
Protein	2545	2597
**ADP**	27	27
Compound	84	44
Ion	3	5
Solvent	234	481
**B-factors (Å^2)**		
Protein	22.0	20.2
ADP	11.7	10.7
Compound	21.2	33.8
Ion	14.5	19.9
Solvent	22.4	34.9
**R.m.s. deviations**		
Bond length(Å)	0.011	0.007
Bond Angle (°)	1.738	1.327

* The numbers in parentheses are for the highest resolution shell.

General Chemistry Methods

Chemicals were purchased from Sigma-Aldrich, Acros, or Alfa Aesar and used without further purification. Anhydrous solvents from Sigma-Aldrich were used without further treatment. Diethylphosphonoacetic acid (**4**) was obtained from Sigma-Aldrich and methylenebisphonic acid triethyl ester (**8**) was synthesised from [ethyloxybenzyloxyphosphorylmethyl]phosphonic acid diethyl ester[5] (see details below). TLC was performed on precoated plates (Merck Aluminum sheets silica 60 F_{254}, art No. 5554). Chromatograms were visualised under UV light and by dipping plates into either phosphomolybdic acid in EtOH or alkaline $KMnO_4$ solution, followed by heating. Flash column chromatography was performed on an ISCO CombiFlash Rf automated flash chromatography system using RediSep Rf disposable flash columns. Ion exchange chromatography was performed on an LKB-Pharmacia Gradifrac medium pressure ion-exchange chromatograph using Q Sepharose Fast Flow resin and a gradient of 0 to 100% 2.0 moldm^{-3} aqueous triethylammonium bicarbonate (TEAB, pH 7.8). Proton ^{1}H NMR, COSY, HMBC and HMQC spectra were recorded on Bruker Avance III (400 MHz and 500 MHz) spectrometers. Proton chemical shifts are reported in ppm (δ) relative to internal tetramethylsilane (TMS, δ 0.0 ppm) or with the solvent reference relative to TMS employed as the internal standard (D_2O: 4.79 ppm). The following abbreviations are used to describe resonances: br, broad; s, singlet; d, doublet; dd, double doublet; q, quartet; m, multiplet; t, triplet. ^{13}C and DEPT spectra were recorded on Bruker Avance III (100 MHz and 126 MHz) spectrometers with complete proton decoupling. Carbon chemical shifts are reported in ppm (δ) relative to TMS with the respective solvent resonance as the internal standard. ^{31}P NMR spectra were recorded on a Bruker Avance III (162 MHz) spectrometer with complete proton decoupling. Phosphorus chemical shifts are reported in ppm (δ) relative to an 85% H_3PO_4 external standard (H_3PO_4, δ 0.0 ppm). Optical rotations were measured at ambient temperature using an Optical Activity Ltd. AA-10 polarimeter, and $[\alpha]_D$ values are given in 10^{-1} deg cm^2 g^{-1}. Melting points were determined using a Reichert-Jung Thermo Galen Kofler block or a Stanford Research Systems Optimelt MPA100 automated melting point system and are uncorrected. Microanalysis was carried out at the University of Bath microanalysis service. Mass spectra were recorded at the SERC Mass Spectrometry Service Centre, Swansea, and at the University of Bath on VG Autospec or MicroTOF instruments.

Synthesis of 1D-2,3,4,6-tetra-*O*-benzyl-*myo*-inositol [(–)-3] and determination of its absolute configuration

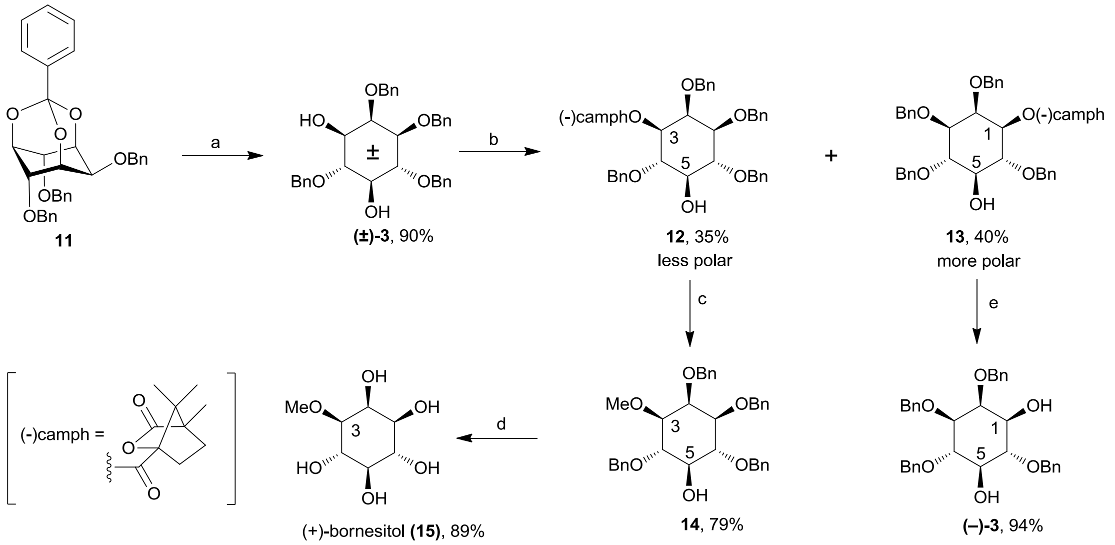

Scheme Synthesis and determination of absolute configuration of **(–)-3**. *Reagents and conditions*: (a) DIBAL-H, CH_2Cl_2, –78℃ to rt; (b) (–)-(1*S*)-camphanic chloride, pyridine, 0℃ to rt; (c) (i) 2-methoxypropene, catalytic PTSA, THF, 0℃ to rt; (ii) LiOH, MeOH, H_2O, THF; (iii) NaH, MeI, DMF, 0℃ to rt; (iv) TFA, CH_2Cl_2, H_2O; (d) H_2, $Pd(OH)_2/C$, EtOH, AcOH.

Diol **(–)-3** was synthesised in three steps from 2,4,6-tri-*O*-benzyl *myo*-inositol orthobenzoate (**11**)[6]. Regioselective reduction[7] of **11** using 3.5 equivalents of DIBAL-H gave racemic diol **(±)-3**. To obtain the required enantiomer of **3**, we applied a previously reported[8] optical resolution of this diol using regioselective formation of diastereoisomeric monocamphanate esters. Thus, reaction of **(±)-3** with 1.1 equivalents of (–)-(1*S*)-camphanic chloride in pyridine gave monocamphanate esters **12** and **13**. Separation of these esters by flash chromatography and hydrolysis of the more polar ester **13**, previously identified as the 1-*O*-(–)-camphanate ester[8] gave **(–)-3**. However, the measured specific rotation of this material {$[\alpha]_D^{20}$ = –9.6, (*c* = 6, $CHCl_3$)} was opposite to that previously reported for D-2,3,4,6-tetra-*O*-benzyl-*myo*-inositol [+10, (*c* = 1, $CHCl_3$)].[8] It was therefore necessary to establish the absolute configurations of camphanates **12** and **13** by an independent route. Thus, transient protection of the free 5-OH group in **12** as a methoxyisopropylidene (MIP) acetal,[9] followed by alkaline hydrolysis of the camphanate ester, methylation of the exposed OH group with MeI/NaH, then mild acid hydrolysis gave **14**. Hydrogenolysis of the benzyl ethers in **14** yielded (+)-bornesitol (3-*O*-methyl-inositol, **15**), identifying **12** as the 3-*O*-(–)-camphanate ester. This unambiguously identifies diastereoiomer **13** as the 1-*O*-(–)-camphanate ester and therefore confirms the identity of **(–)-3** as 1D-2,3,4,6-tetra-*O*-benzyl-*myo*-inositol.

DL-1,2,4,6-tetra-*O*-benzyl-*myo*-inositol [(±)-3]

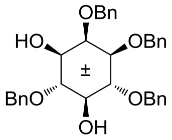

(±)-3

To a solution of 2,4,6-tri-*O*-benzyl-*myo*-inositol orthobenzoate (**11**)[6] (2.15 g, 4.00 mmol) in dry dichloromethane (30 mL) at –78 °C was added a solution of DIBAL-H (14 mL of a 1.0 M solution in hexane, 14 mmol) dropwise under N_2. Stirring was continued overnight, during which time the solution was allowed to warm slowly to room temperature. TLC (EtOAc:light petroleum, 1:2) now showed complete conversion of orthobenzoate **11** (R_f 0.70) into a more polar product (R_f 0.48). The reaction was carefully quenched by adding water (40 mL), and then 1.0 moldm^{-3} aqueous HCl (40 mL) and dichloromethane (100 mL) were added. The organic layer was separated and the aqueous layer was re-extracted with dichloromethane (2 × 50 mL). The combined organic extracts were dried ($MgSO_4$) and concentrated to give an oil (~2.5 g), which was purified by flash chromatography (EtOAc in petroleum ether 0 to 100%) giving **(±)-3** as a colourless oil, which gradually crystallised as a waxy solid (1.95 g, 3.61 mmole, 90%); TLC (EtOAc:light petroleum 1:2): R_f = 0.48; m.p. 83.5–85.5 °C (from diisopropyl ether/light petroleum; ^{1}H NMR ($CDCl_3$, 400 MHz) δ 2.33 (1 H, d, *J* 6.3 Hz, 3-OH), 2.52 (1 H, d, *J* 2.1 Hz, 5-OH), 3.44 (1 H, dd, *J* 9.8, 2.4 Hz, H-1), 3.47 (1 H, ddd, *J* 9.4, 6.3, 2.4 Hz, H-3), 3.52 (1 H, td, *J* 9.4, 2.1 Hz, H-5), 3.69 (1 H, t, *J* 9.4 Hz, H-4), 3.90 (1 H, t, *J* 9.4 Hz, H-6), 4.04 (1 H, t, *J* 2.5 Hz, H-2), 4.68 (2 H, br s, OC*H*$_2$Ph), 4.73 (1 H, d, J_{AB} 11.6 Hz, OC*H*HPh), 4.76 (1 H, d, J_{AB} 11.2 Hz, OC*H*HPh), 4.79 (1 H, d, J_{AB} 11.3 Hz, OC*H*HPh), 4.89 (1 H, d, J_{AB} 11.3 Hz, OC*H*HPh), 4.98 (1 H, d, J_{AB} 11.2 Hz, OC*H*HPh), 4.99 (1 H, d, J_{AB} 11.6 Hz, OC*H*HPh) and 7.25–7.38 (20 H, m, Ph); ^{13}C NMR ($CDCl_3$, 100 MHz) δ 72.20 (C-3), 72.70 (O*C*H$_2$Ph), 74.76 (O*C*H$_2$Ph), 74.92 (O*C*H$_2$Ph), 75.01 (C-5), 75.01 (O*C*H$_2$Ph), 75.47 (O*C*H$_2$Ph), 77.07 (C-2), 80.08 (C-1), 81.26 (C-6), 81.66 (C-4), 127.60, 127.65, 127.74, 127.77, 127.99 and 128.03 (20 × Ph CH), 138.08, 138.66, 138.67 and 138.69 (4 × *ipso-C* of Ph); HRMS (*m/z*) [M-H]$^-$ calcd. for $C_{34}H_{35}O_6$, 539.2439; found 539.2461; Anal. calcd for $C_{34}H_{36}O_6$; C 75.53, H, 6.71; found C 75.39, H, 6.66.

D-1,2,4,6-tetra-*O*-benzyl-*myo*-inositol 3-*O*-[(–)-(1*S*)-camphanoate)] (12) and D-2,3,4,6-tetra-*O*-benzyl-*myo*-inositol 1-*O*-[(–)-(1*S*)-camphanoate)] (13)

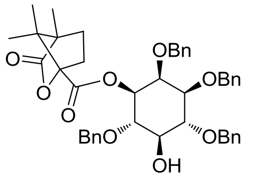

12

and

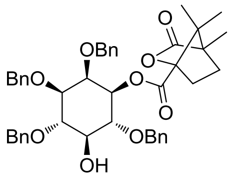

13

To a stirred solution of racemic diol **(±)-3** (1.53 g, 2.83 mmol) in dry pyridine (10 mL) at 0 °C was added (–)-(1*S*)-camphanic chloride (700 mg, 3.23 mmol) as a solid in small portions over 1 min. Stirring was continued overnight, during which time the solution was allowed to warm slowly to room temperature. TLC (dichloromethane:ethyl acetate 10:1) showed conversion of diol (R_f 0.54) into two less polar products. The solution was concentrated *in vacuo* and the residue was taken up in diethyl ether (100 mL). This solution was washed with 1.0 moldm^{-3} aqueous HCl (2 × 50 mL) followed by brine (50 mL), then dried ($MgSO_4$) and concentrated to give a solid residue, which contained the two diastereiomeric monocamphanate esters **12** and **13**, together with a trace of unreacted diol **(±)-3**. An initial purification step by flash chromatography (EtOAc in petroleum ether, 0 to 50%) was used to remove this unreacted diol, which could otherwise cause difficulties with the isolation of the more polar monocamphanate ester. The monocamphanate esters were then separated by flash chromatography (ethyl acetate in dichloromethane, 0 to 10%), giving first the 3-*O*-camphanate ester **12** (719 mg, 1.00 mmol, 35%), followed by the 1-*O*-camphanate ester **13** (811 mg, 1.13 mmol, 40%).

Data for 3-*O*-camphanate ester (12); R_f 0.30, ethyl acetate:dichloromethane 3:97; crystals from ethyl acetate/light petroleum, m.p. 163.5–165 °C; $[\alpha]_D^{20}$ = +21, (*c* = 1, $CHCl_3$); ^{1}H NMR ($CDCl_3$, 400 MHz) δ 0.88 (3 H, s, camph CH_3), 1.00 (3 H, s, camph CH_3), 1.08 (3 H, s, camph CH_3), 1.58–1.66 (1 H, m, camph C*H*H), 1.81–1.89 (2 H, m, 2 × camph C*H*H), 2.25–2.33 (1 H, m, camph C*H*H), 2.48 (1 H, d, *J* 2.3 Hz, 5-OH), 3.56 (1 H, dd, *J* 9.7, 2.3 Hz, H-1), 3.63 (1 H, td, *J* 9.3 Hz, 2.3 Hz, H-5), 3.94 (1 H, t, *J* 9.4 Hz, H-6), 4.04 (1 H, t, *J* 9.3 Hz, H-4), 4.15 (1 H, t, *J* 2.3 Hz, H-2), 4.68 (1 H, d, J_{AB} 11.5 Hz, OC*H*HPh), 4.69 (2 H, br s, OCH_2Ph), 4.74 (1 H, d, J_{AB} 11.5 Hz, OC*H*HPh), 4.76 (1 H, d, J_{AB} 11.3 Hz, OC*H*HPh), 4.89 (2 H, d, J_{AB} 11.4 Hz, 2 × OC*H*HPh), 4.95 (1 H, buried, H-3), 4.96 (1 H, d, J_{AB} 11.1 Hz, OC*H*HPh), 7.24–7.40 (20 H, Ph); ^{13}C NMR ($CDCl_3$, 100 MHz) δ 9.66 (camph CH_3), 16.61 (camph CH_3), 16.69 (camph CH_3), 28.89 (camph CH_2), 30.72 (camph CH_2), 54.22 (camph quaternary C), 54.77 (camph quaternary C), 72.82 (OCH_2Ph), 74.68 (OCH_2Ph), 74.71 and 75.06 (C-1 and C-5), 75.05 (OCH_2Ph), 75.55 (OCH_2Ph), 75.98 (C-2), 78.64 (C-4), 80.65 and 80.84 (C-1 and C-6), 90.84 (quaternary C), 127.45 (4 C), 127.53 (3 C),

127.63 (1 C), 127.77 (1 C), 127.80 (1 C), 128.02 (2 C), 128.32 (2 C), 128.33 (2 C), 128.47 (2 C) and 128.51 (2 C) (20 × Ph CH), 137.92, 138.29, 138.45 and 138.54 (4 × *ipso-C* of Ph), 167.23 (C=O), 177.96 (C=O); HRMS (*m/z*) $[M+H]^+$ calcd. for $C_{44}H_{48}O_9$ 721.3371; found 721.3405; Anal. calcd for $C_{44}H_{48}O_9$; C 73.31, H, 6.71; found C 73.24, H, 6.82.

Data for 1-*O*-camphanate ester (13); R_f 0.22, ethyl acetate:dichloromethane 3:97; crystals from acetone/light petroleum, m.p. 167–169.5 °C; $[\alpha]_D^{20}$ = –27, *c* = 1, $CHCl_3$); ^1H NMR ($CDCl_3$, 400 MHz) δ 0.87 (3 H, s, camph CH_3), 0.95 (3 H, s, camph CH_3), 1.08 (3 H, s, camph CH_3), 1.62–1.69 (1 H, m, camph C*H*H), 1.82–1.95 (2 H, m, 2 × camph C*H*H), 2.26–2.34 (1 H, m, camph C*H*H), 2.47 (1 H, d, *J* 2.2 Hz, 5-OH), 3.55 (1 H, dd, *J* 9.7, 2.0 Hz, H-3), 3.62 (1 H, td, *J* 9.1 Hz, 2.2 Hz, H-5), 3.95 (1 H, t, *J* 9.4 Hz, H-4), 4.05 (1 H, t, *J* 9.4 Hz, H-6), 4.22 (1 H, br s, H-2), 4.65–4.77 (3 H, m, 3 × OC*H*HPh), 4.68 (2 H, br s, OCH_2Ph), 4.88 (1 H, d, J_{AB} 11.3 Hz, OC*H*HPh), 4.89 (1 H, dd, *J* 10.1, 2.4 Hz, H-1), 4.95 (1 H, d, J_{AB} 11.5 Hz, OC*H*HPh), 4.96 (1 H, d, J_{AB} 11.2 Hz, OC*H*HPh), 7.24–7.40 (20 H, Ph); ^{13}C NMR ($CDCl_3$, 100 MHz) δ 9.63 (camph CH_3), 16.56 (camph CH_3), 16.61 (camph CH_3), 28.90 (camph CH_2), 30.89 (camph CH_2), 54.18 (camph quaternary C), 54.82 (camph quaternary C), 72.81 (OCH_2Ph), 74.69 (OCH_2Ph), 74.75 (OCH_2Ph), 75.01 (1 C) and 75.17 (2 C) (C-1, C-2 and C-5), 75.56 (OCH_2Ph), 78.50 (C-6), 80.69 and 80.77 (C-3 and C-4), 90.87 (quaternary C), 127.46 (2 C), 127.51 (2 C), 127.57 (1 C), 127.61 (3 C), 127.77 (1 C), 127.81 (1 C), 128.03 (2 C), 128.32 (2 C), 128.37 (2 C), 128.47 (2 C) and 128.51 (2 C) (20 × Ph CH), 137.88, 138.27, 138.41 and 138.54 (4 × *ipso-C* of Ph), 167.42 (C=O), 177.99 (C=O); HRMS (*m/z*) $[M+Na]^+$ calcd. for $C_{44}H_{48}O_9$ 743.3196; found 743.3216; Anal. calcd for $C_{44}H_{48}O_9$; C 73.31, H, 6.71; found C 73.22, H, 6.77.

Determination of Absolute Configurations of Monocamphanate Esters 12 and 13

D-1,2,4,6-tetra-*O*-benzyl-3-*O*-methyl-*myo*-inositol (14)

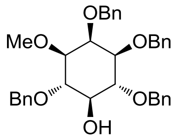

14

To a solution of the less polar monocamphanate **12** (200 mg, 0.277 mmol) in dry THF (4 mL) at 0 °C was added a strictly catalytic amount of PTSA (2 mg), followed by 2-methoxypropene (0.60 mL, 0.45 g, 6.2 mmol). After 10 min, the cooling bath was removed and the solution was allowed to reach room temperature over 1 hour. TLC (EtOAc:light petroleum 1:3) showed conversion of **12** (R_f 0.36) into a less polar product (R_f 0.54). The solution was stirred at room temperature and a solution of $LiOH.H_2O$ (118 mg, 2.8 mmol) in deionised water (2 mL) and methanol (2 mL) was added. After 2 h, TLC (EtOAc:light petroleum 1:3) showed that

the camphanate ester had been cleaved, giving a slightly more polar product (R_f 0.40). The volatile solvents were removed by evaporation under reduced pressure, and then more deionised water (20 mL) was added, followed by dichloromethane (20 mL). The organic layer was separated and the aqueous layer was re-extracted with more dichloromethane (20 mL). The combined organic layers were dried ($MgSO_4$) and concentrated. The residue was re-dissolved in dry DMF (5 mL) and sodium hydride (110 mg of a 60% suspension in oil, 2.8 mmol) was added. The suspension was stirred at rt for 5 min, then cooled to 0 °C under N_2. Methyl iodide (137 μL, 2.2 mmol) was added dropwise and stirring was continued at 0 °C for a further 30 min before the cooling bath was removed and the solution was stirred at rt for a further 1 h. The reaction was quenched by careful addition of water (25 mL) and the resulting suspension was extracted with diethyl ether (2 × 30 mL). TLC (EtOAc:light petroleum 1:3) showed conversion of the alcohol into a less polar product (R_f 0.70). The combined organic extracts were concentrated and the residue was taken up in dichloromethane (10 mL) before adding TFA (4 drops) and water (1 drop). TLC after 30 min showed complete conversion into a more polar product (R_f 0.36). The solution was concentrated and the residue was purified by flash chromatography (EtOAc in petroleum ether 0 to 50%) to give **14** as a white solid (122 mg, 0.220 mmol, 79% over 4 steps); TLC (EtOAc:light petroleum 1:3): R_f = 0.36; crystals from ethyl acetate/light petroleum, m.p. 114–115.5 °C, $[\alpha]_D^{20}$ = –7.4, *c* = 1, $CHCl_3$); ^{1}H NMR ($CDCl_3$, 400 MHz) δ 2.46 (1 H, d, *J* 2.1 Hz, 5-OH), 3.09 (1 H, dd, *J* 9.7, 2.3 Hz, H-3), 3.36 (1 H, dd, *J* 9.7, 2.3 Hz, H-1), 3.41 (3 H, s, CH_3O), 3.51 (1 H, td, *J* 9.2, 2.1 Hz, H-5), 3.85 (1 H, t, *J* 9.5 Hz, H-4), 3.94 (1 H, t, *J* 9.5 Hz, H-6), 4.10 (1 H, t, *J* 2.3 Hz, H-2), 4.63, 4.67 (2 H, AB system, J_{AB} 11.8 Hz), 4.76 (1 H, d, J_{AB} 11.2 Hz, OC*H*HPh), 4.80 (1 H, d, J_{AB} 11.1 Hz, OC*H*HPh), 4.86, 4.89 (2 H, AB system, J_{AB} 12.0 Hz), 4.91 (1 H, d, J_{AB} 11.1 Hz, OC*H*HPh), 4.92 (1 H, d, J_{AB} 11.2 Hz, OC*H*HPh), and 7.24–7.44 (20 H, m, Ph); ^{13}C NMR ($CDCl_3$, 100 MHz) δ 58.22 (CH_3O), 72.74 (OCH_2Ph), 73.62 (C-2), 74.17 (OCH_2Ph), 75.01 (C-5), 75.22(OCH_2Ph), 75.45 (OCH_2Ph), 80.02, 81.10 and 81.14 (C-1, C-4 and C-6), 82.89 (C-3), 127.36 (1 C), 127.52 (2 C), 127.60 (1 C), 127.62 (2 C), 127.72 (2 C), 127.92 (2 C), 128.03 (2 C), 128.15 (2 C), 128.38 (2 C), and 128.41 (4 C) (20 × Ph CH), 138.36, 138.86, 138.96 and 139.00 (4 × *ipso-C* of Ph); HRMS (*m*/*z*) $[M+Na]^+$ calcd. for $C_{35}H_{38}O_6$, 577.2561; found 577.2592; Anal. calcd for $C_{35}H_{38}O_6$; C 75.79, H, 6.91; found C 75.69, H, 6.95.

D-3-*O*-Methyl-*myo*-inositol [(+)-bornesitol, 15]

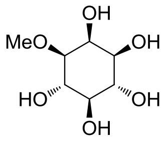

15

To a solution of **14** (110 mg, 0.198 mmol) in ethanol (10 mL) and acetic acid (2 mL) was added palladium hydroxide on activated charcoal (20%, 50% water, 50 mg). The suspension was stirred vigorously under an atmosphere of H_2 (balloon) for 18 h. The catalyst was removed by filtration through a PTFE filter, giving a colourless solution, which was concentrated under reduced pressure. The residue was re-dissolved in deionised water and lyophilised to give **15** as a fluffy white solid (34.1 mg, 0.176 mmol, 89%); TLC (acetone:water 5:1): R_f = 0.38; $[\alpha]_D^{20}$ = +31, (*c* = 0.7, H_2O), Lit.[10] $[\alpha]_D^{20}$ = +31.9 (*c* = 1, H_2O)[10]; crystals from ethanol/methanol m.p. 204–206 °C, Lit.[10] 205–207 °C; ^{1}H NMR (D_2O, 400 MHz) δ 3.18 (1 H, dd, *J* 9.9, 2.5 Hz, H-3), 3.27 (1 H, t, *J* 9.4 Hz, H-5), 3.43 (3 H, s, CH_3O), 3.49 (1 H, dd, *J* 10.0, 2.6 Hz, H-1), 3.61 (1 H, t, *J* 10.0 Hz, H-6), 3.64 (1 H, t, *J* 10.0 Hz, H-4), 4.30 (1 H, t, *J* 2.4 Hz, H-2); ^{13}C NMR (D_2O, 100 MHz) δ 56.61(CH_3O), 67.55 (C-2), 70.99 (C-1), 71.55 (C-4), 72.18 (C-6), 74.35 (C-5) and 80.42 (C-3); HRMS (*m/z*) $[M+Na]^+$ calcd. for $C_7H_{14}O_6$, 217.0683; found 217.0674.

D-2,3,4,6-tetra-*O*-benzyl-*myo*-inositol [(–)-3]

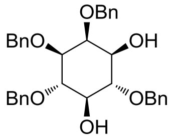

(–)-3

A suspension of the more polar monocamphanate (**13**, 400 mg, 0.555 mmol) in methanol (20 mL) and sodium hydroxide (one small pellet, 180 mg, 4.5 mmol) was heated at reflux for 1 h. The resulting clear solution was allowed to cool, neutralised by addition of solid CO_2 pellets, and then concentrated. The residue was partitioned between water and dichloromethane (40 mL each) and the organic layer was separated, dried ($MgSO_4$) and concentrated. Purification of the residue by flash chromatography (EtOAc in petroleum ether 0 to 100%) gave diol **(–)-3** as a colourless oil (281 mg, 0.520 mmol, 94%); $[\alpha]_D^{20}$ = –9.6, (*c* = 6, $CHCl_3$), Lit.[8] +10, (*c* = 1, $CHCl_3$); other data were identical to those obtained for the racemic tetraol **(±)-3**.

D-2,3,4,6-tetra-*O*-benzyl-*myo*-inositol 1,5-di-*O*-(diethyloxyphosphorylacetate) (5)

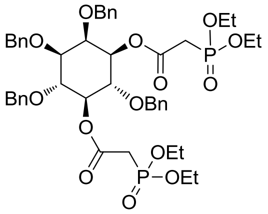

5

To a solution of 1D-2,3,4,6-tetra-*O*-benzyl-*myo*-inositol [**(–)-3**, 220 mg, 0.407 mmole] and EDAC hydrochloride (203 mg, 1.06 mmole) in dry dichloromethane (8 mL) under N_2 at RT was added diethylphosphonoacetic acid (**4**) (0.2 mL, 244 mg, 1.24 mmole). The solution was stirred for 4 h. TLC (EtOAc) showed complete conversion of alcohol (R_f 0.88) into a more polar product (R_f 0.50). Dichloromethane (30 mL) was added. The solution was washed with sat. aq. $NaHCO_3$ and 1.0 moldm^{-3} aqueous HCl (40 mL each), dried ($MgSO_4$) and concentrated to give an oil. Purification by flash chromatography on silica (ethyl acetate in light petroleum, 0 to 100%) gave **5** as a colourless oil (332 mg, 0.370 mmole, 91%); TLC (EtOAc:light petroleum 2:1): R_f = 0.22, $[\alpha]_D^{20}$ = –17, *c* = 1, $CHCl_3$); ^{1}H NMR ($CDCl_3$, 500 MHz) δ 1.18–1.27 (12 H, m, 4 × OCH_2CH_3), 2.64–2.79 (4 H, m, 2 × C(O)C*H*$_2$P), 3.58 (1 H, dd, *J* 9.7, 2.2 Hz, H-3), 3.97–4.12 (10 H, m, H-4, H-6 and 4 × OC*H*$_2$CH$_3$), 4.16 (1 H, t, *J* 2.2 Hz, H-2), 4.65 (2 H, br s, OC*H*$_2$Ph), 4.65–4.74 (4 H, m, 4 × OC*H*HPh), 4.80–4.88 (3 H, m, H-1 and 2 × OC*H*HPh), 5.17 (1 H, t, *J* 9.5 Hz, H-5) and 7.23–7.39 (20 H, m, Ph); ^{13}C NMR ($CDCl_3$, 100 MHz) δ 16.21–16.34 (with $^3J_{CP}$ couplings, 4 × $POCH_2CH_3$), 34.00 ($^1J_{CP}$ 135 Hz, CH_2P), 34.02 ($^1J_{CP}$ 136 Hz, CH_2P), 65.58–62.80 (with $^2J_{CP}$ couplings, 4 × PO*C*H$_2$CH$_3$), 73.01 (O*C*H$_2$Ph), 74.57 (O*C*H$_2$Ph), 74.71, 75.06 and 75.42 (C-1, C-2 and C-5), 74.90 (O*C*H$_2$Ph), 75.10 (O*C*H$_2$Ph), 77.21 (C-6), 78.73 (C-4), 80.59 (C-3), 127.46, 127.48, 127.59, 127.64, 127.74, 127.77, 127.82, 128.28 and 128.44 (20 × Ph CH), 137.77, 138.48, 138.52 and 138.54 (4 × *ipso-C* of Ph), 164.71 ($^2J_{CP}$ 5.7 Hz, C=O), 165.28 ($^2J_{CP}$ 5.7 Hz, C=O); ^{31}P NMR ($CDCl_3$, 162 MHz) δ 19.07 (1 P), 19.26 (1 P); HRMS (*m/z*) $[M–H]^-$ calcd. for $C_{46}H_{58}O_{14}P_2$; 895.3229; found 895.3199.

D-*myo*-inositol 1,5-di-*O*-(diethyloxyphosphorylacetate) (6)

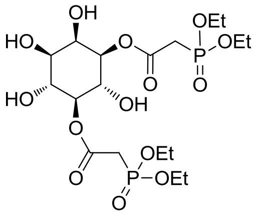

6

To a solution of **5** (139 mg, 0.155 mmol) in MeOH (7 mL) and deionised water (1 mL) was added palladium hydroxide on activated charcoal (20%, 50% water, 70 mg). The suspension was stirred vigorously under an atmosphere of H_2 (balloon) for 48 h. The catalyst was

removed by filtration through a PTFE filter, giving a colourless solution, which was concentrated under reduced pressure. The residue (82 mg) was purified by flash chromatography (methanol in dichloromethane, 0 to 20%) to give **6** as a colourless glassy solid (66 mg, 0.123 mmole, 79 %); TLC (dichloromethane:methanol 10:1): R_f = 0.16; $[\alpha]_D^{20}$ = –18.7, *c* = 1.3, $CHCl_3$); ^{1}H NMR ($CDCl_3$, 400 MHz) *δ* 1.33–1.37 (12 H, m, 4 × OCH_2CH_3), 2.99–3.16 (4 H, m, 2 × C(O)C*H*$_2$P), 3.60 (2 H, brs, H-3 and 3-OH), 3.94 (1 H, brt, *J* ~9 Hz, H-4), 4.12–4.23 (9 H, m, H-6 and 4 × OC*H*$_2$CH$_3$), 4.25 (1 H, brs, H-2), 4.37 (1 H, br s, 4-OH), 4.47 (1 H, br s, 2-OH), 4.73 (1 H, br d, *J* 3.1 Hz, 6-OH), 4.81 (1 H, dd, *J* 10.0, 2.4 Hz, H-1) 4.88 (1 H, t, *J* 9.6 Hz, H-5); ^{13}C NMR ($CDCl_3$, 100 MHz) *δ* 16.23 and 16.26 (4 × POCH$_2$*C*H$_3$), 34.73 ($^1J_{CP}$ 130.8 Hz, CH$_2$P), 34.93 ($^1J_{CP}$ 130.1 Hz, CH$_2$P), 63.27–63.39 (overlapping signals with $^2J_{CP}$ couplings, 4 × PO*C*H$_2$CH$_3$), 68.64 (C-6), 69.21 (C-2), 70.76 (C-4), 71.38 (C-3), 75.63 (C-1), 77.69 (C-5), 165.28 ($^2J_{CP}$ 6.1 Hz, C=O), 165.53 ($^2J_{CP}$ 5.9 Hz, C=O); ^{31}P NMR ($CDCl_3$, 162 MHz) *δ* 21.19 (1 P), 21.78 (1 P); HRMS (*m/z*) $[M–H]^–$ calcd. for $C_{18}H_{34}O_{14}P_2$; 535.1351; found 535.1348.

D-*myo*-inositol 2,3,4,6-tetrakis(dibenzylphosphate) 1,5-di-*O*-(diethyloxyphosphorylacetate) (7)

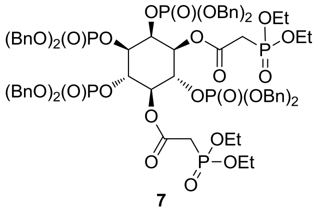

7

To a stirred suspension of 5-phenyltetrazole (98 mg, 0.672 mmol) in dry dichloromethane (2 mL) under N_2 at room temperature was added bis(benzyloxy)diisopropylaminophosphine (0.2 mL, 0.6 mmol). After 30 min, the resulting clear solution was added to the tetraol **6** (60 mg 0.112 mmol). The mixture was stirred under N_2 at room temperature for 2 h and then cooled to –78 °C, before MCPBA (70%, 221 mg, 0.896 mmol) was added. The mixture was allowed to warm to room temperature and then diluted with EtOAc (30 mL). The clear, colourless solution was washed with 10% aq. Na_2SO_3 solution (2 × 30 mL), dried over $MgSO_4$ and concentrated. The residue was purified by flash chromatography (acetone in dichloromethane 0 to 40%) to give **7** as a colourless oil (146 mg, 0.093 mmole, 83%); TLC (dichloromethane:acetone 2:1): R_f = 0.40; $[\alpha]_D^{20}$ = +3, *c* = 3, $CHCl_3$); ^{1}H NMR ($CDCl_3$, 400 MHz) *δ*1.19 (3 H, t, *J* 7.1 Hz, OCH$_2$C*H*$_3$), 1.20 (3 H, t, *J* 7.1 Hz, OCH$_2$C*H*$_3$), 1.24 (3 H, t, *J* 7.1 Hz, OCH$_2$C*H*$_3$), 1.26 (3 H, t, *J* 7.1 Hz, OCH$_2$C*H*$_3$), 2.71, 2.84 (2 H, ABX system. J_{AB} 16.2 Hz, J_{HP} 20.0 Hz, C(O)C*H*$_2$P) 3.02, 3.09 (2 H, ABX system. J_{AB} ~ J_{HP} ~ 18 Hz, C(O)C*H*$_2$P), 3.98–4.13 (8 H, m, 4 × OC*H*$_2$CH$_3$), 4.34–4.40 (1 H, m, H-3), 4.81–5.15 (19 H, m, H-1, H-4, H-6 and 8 × POCH$_2$Ph), 5.29 (1 H, t, *J* 9.5 Hz, H-5), 5.32 (1 H, dt, *J* ~9, ~2 Hz, H-2), 7.14–7.39 (40 H, m, 8 × Ph); ^{13}C NMR

(CDCl$_3$, 100 MHz) δ 16.28 and 16.34 (4 × POCH$_2$*C*H$_3$), 32.76 ($^1J_{CP}$ 149.4 Hz, CH$_2$P), 32.96 ($^1J_{CP}$ 142.5 Hz, CH$_2$P), 62.32 ($^2J_{CP}$ 6.1 Hz, PO*C*H$_2$CH$_3$), 62.36 ($^2J_{CP}$ 6.1 Hz, PO*C*H$_2$CH$_3$), 62.52 ($^2J_{CP}$ 6.3 Hz, PO*C*H$_2$CH$_3$), 62.74 ($^2J_{CP}$ 6.2 Hz, PO*C*H$_2$CH$_3$), 69.61–70.13 (overlapping signals with $^2J_{CP}$ couplings, Ph*C*H$_2$OPO), 71.51 (C-5), 73.40 (C-3), 74.22 and 74.65 (C-1, C-2, C-4 and C-6), 127.84–128.64 (*C*H of Ph), 135.35–135.58 (overlapping signals with $^3J_{CP}$ couplings, *ipso-C* of *Ph*CH$_2$OP), 165.20 ($^2J_{CP}$ 3.1 Hz, C=O), 165.89 (broad, C=O); ^{31}P NMR (CDCl$_3$, 162 MHz) δ –2.05 [1 P, (BnO)$_2$*P*(O)O], –1.52 [1 P, (BnO)$_2$*P*(O)O], –1.40 [1 P, (BnO)$_2$*P*(O)O], –1.29 [1 P, (BnO)$_2$*P*(O)O], 18.73 [1 P, (EtO)$_2$*P*(O)CH$_2$], 19.47 [1 P, (EtO)$_2$*P*(O)CH$_2$]; HRMS (*m/z*) [M+Na]$^+$ calcd. for $C_{74}H_{86}O_{26}P_6$; 1599.3725; found 1599.3757.

D-*myo*-inositol 2,3,4,6-tetrakisphosphate-1,5-bis-*O*-phosphorylacetate (1)

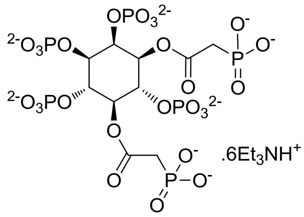

1

A stirred solution of **7** (115 mg, 73 μmole) in dry dichloromethane (2 mL) was cooled to 0 °C under N$_2$ and trimethylsilyl bromide (1 mL) was added dropwise over 5 min. The solution was allowed to warm gradually to room temperature, and stirring was continued for 48 h. The solution was concentrated and methanol (5 mL) was added to the residue. The resulting colourless solution was stirred at room temperature for a further 1 h, then concentrated to give a white gum. The gum was washed with diethyl ether (3 × 3 mL), then taken up in 1.0 moldm^{-3} TEAB (10 mL, pH 7.6) to give a cloudy suspension. This suspension was washed with diethyl ether (3 × 10 mL) to give a colourless, clear solution. This solution was concentrated under reduced pressure, re-dissolved in methanol and concentrated again. This cycle was repeated several times until a clear, colourless, glassy solid remained, which was finally re-dissolved in de-ionised water and lyophilised to give the triethylammonium salt of **1** as a brittle colourless solid (89 mg, 61 μmole, 84 %); $[\alpha]_D^{20}$ = –4.5, *c* = 1, MeOH); ^{1}H NMR (D$_2$O, 400 MHz) δ 1.24 (~54 H, t, *J* 7.3 Hz, CH$_3$ of TEA$^+$), 2.85–3.02 (4 H, m, 2 × C*H*$_2$P), 3.16 (~36 H, q, *J* 7.3 Hz, CH$_2$ of TEA$^+$), 4.31 (1 H, broad t, *J* 9.9 Hz, H-3), 4.56 (1 H, q, *J* 9.5 Hz, H-4), 4.64 (1 H, q, *J* 9.7 Hz, H-6), approx. 4.8 (1 H, buried by HDO, H-2), 5.05 (1 H, broad d, *J* 10.2 Hz, H-1), 5.14 (1 H, t, *J* 9.5 Hz, H-5); ^{13}C NMR (D$_2$O, 100 MHz) δ 8.23 (CH$_3$ of TEA$^+$), 37.06 ($^1J_{CP}$ 117.9 Hz, CH$_2$P), 37.42 ($^1J_{CP}$ 118.5 Hz, CH$_2$P), 46.60 (CH$_2$ of TEA$^+$), 71.69 (C-1), 72.72 (C-6), 73.20 (C-3), 73.62 (C-5), 73.81 (C-2), 74.74 (C-4), 169.50 ($^2J_{CP}$ 6.3 Hz, C=O), 169.57 ($^2J_{CP}$ 6.3 Hz, C=O); ^{31}P NMR (D$_2$O, 162 MHz) δ –0.73 (1 P, phosphate P), –0.55 (1 P, phosphate P), 0.05 (1 P,

phosphate P), 0.12 (1 P, phosphate P), 11.60 (1 P, phosphonate P), 12.20 (1 P, phosphonate P); ^{31}P NMR (CD_3OD, 162 MHz) δ –0.67 (1 P, phosphate P), 0.43 (1 P, phosphate P), 0.55 (1 P, phosphate P), 1.12 (1 P, phosphate P), 11.60 (1 P, phosphonate P), 12.26 (1 P, phosphonate P); HRMS (*m/z*) $[M]^-$ calcd. for $C_{10}H_{21}O_{26}P_6$; 742.8752; found 742.8757.

Methylenebisphosphonic acid triethyl ester (8)

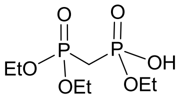

8

To a solution of [ethyloxybenzyloxyphosphorylmethyl]phosphonic acid diethyl ester[5] (1.77 g, 5.05 mmol) in methanol (30 mL) was added acetic acid (5 drops) and palladium hydroxide on activated charcoal (20%, 50% water, 180 mg). The suspension was stirred vigorously under an atmosphere of H_2 (balloon) for 2 days. TLC (ethyl acetate/methanol 10:1) showed that hydrogenolysis was complete with total conversion of starting material (R_f 0.48) into a highly polar product (R_f ~ 0, no UV activity, stain $KMnO_4$). The catalyst was removed by filtration through a PTFE syringe filter and the solution was concentrated and dried under vacuum to give triethyl ester **8** as a colourless oil (1.28 g, 4.92 mmol, 97%; ^{1}H NMR ($CDCl_3$, 400 MHz) δ 1.35 (9 H, t, *J* 7.1 Hz, 3 × C*H*$_3$CH$_2$OP), 2.53 (2 H, t, $^2J_{HP}$ 21.2 Hz, PCH$_2$P), 4.13–4.23 (6 H, m, 3 × CH$_3$C*H*$_2$OP), 11.37 (1 H, broad s, P(O)O*H*); ^{13}C NMR ($CDCl_3$, 100 MHz) δ 16.30 ($^3J_{CP}$ 6.3 Hz, 3 × *C*H$_3$CH$_2$OP), 25.54 ($^1J_{CP}$ 136.8 Hz, P*C*H$_2$P), 62.20 (1 C, $^2J_{CP}$ 6.3 Hz, CH$_3$*C*H$_2$OP), 62.82 (2 C, $^2J_{CP}$ 6.3 Hz, CH$_3$*C*H$_2$OP); ^{31}P NMR ($CDCl_3$, 162 MHz) δ 19.57, 19.85 (2 P, AB system, $^2J_{PP}$ 6.5 Hz); HRMS (*m/z*) $[M]^-$ calcd. for $C_7H_{18}O_6P_2$; 259.0500; found 259.0487; $[M + Na]^+$ calcd. for $C_7H_{18}O_6P_2$; 283.0476; found 283.0475.

D-1,5-Di-*O*-[[bis(ethyloxy)phosphorylmethyl]ethyloxyphosphoryl]-2,3,4,6-tetra-*O*-benzyl-*myo*-inositol (9)

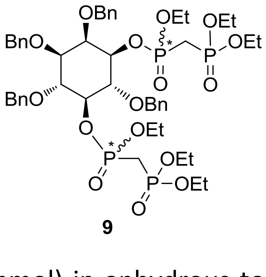

9

To a solution of **8** (670 mg, 2.58 mmol) in anhydrous toluene (4 mL) under N_2 at 0 °C was added oxalyl chloride (0.9 mL, 10 mmol) followed by a catalytic amount of DMF (< 5µL). Evolution of gas was observed on adding the DMF. The solution was stirred at 0 °C for 30 min and then concentrated under reduced pressure to give the crude phosphonochloridate as an oily residue. Diol **(–)-3** (150 mg, 0.277 mmol), dried by dissolving in acetonitrile and

repeatedly concentrating the solution under reduced pressure) was dissolved in anhydrous dichloromethane (2 mL) and cooled to 0 °C. DIPEA (1.0 mL, 5.7 mmol) was added, followed by a solution of the crude phosphonochloridate in anhydrous dichloromethane (3 mL), added dropwise over 5 min. The cooling bath was removed and the solution was stirred at room temperature for 24 h. TLC showed total consumption of diol (R_f 0.74 in ethyl acetate) and the appearance of more polar products; monophosphonylated material (major product, overlapping spots, R_f ~0.3 in ethyl acetate; mixture of diastereoisomers) and bisphosphonylated material **9** (minor product, R_f ~0.05 streak in ethyl acetate; apparently two spots R_f 0.30 and 0.36 in ethyl acetate/methanol 20:1; mixture of diastereoisomers). The reaction mixture was left at room temperature for a further 24 h, but no further reaction occurred, as judged by TLC. The solution was concentrated to give a brown oil, which was taken up in ethyl acetate (20 mL), washed with 1.0 moldm^{-3} HCl, sat aq, $NaHCO_3$ and brine (20 mL each), dried ($MgSO_4$) and concentrated. The residue was purified by flash chromatography (4 g silica, eluting with ethyl acetate, then methanol in ethyl acetate, 0 to 10%) to give first monophosphonylated material as a colourless oil (mixture of diastereoisomers, 169 mg) and then **9** as a yellow oil (mixture of diastereosisomers, 43 mg, 0.042 mmol, 15%) Further **9** could be obtained by repeating the above sequence using the monophosphonylated material (i.e. recycling the major product). After one round of recycling, **9** was obtained in a combined yield of 84 mg, 0.082 mmol, 30%; TLC apparently two spots, R_f 0.30 and 0.36 in ethyl acetate/methanol 20:1; ^{1}H NMR ($CDCl_3$, 500 MHz) δ 0.92–1.29 (18 H, m, 6 × OCH_2CH_3), 2.16–2.37 (4 H, m, 2 × PCH_2P), 3.53–3.60 (1 H, m, H-3 in four diastereosiomers), 3.84–4.22 (14 H, m, 6 × OCH_2CH_3, H-4 and H-6), 4.33–4.53 (3 H, m, H-1, H-2 and H-5 in four diastereosiomers), 4.57–5.09 (8 H, overlapping AB systems, 4×OCH_2Ph), 7.22–7.45 (20 H, Ph); ^{13}C NMR ($CDCl_3$, 126 MHz) δ 15.82–16.37 (6 × $POCH_2CH_3$), 23.73–27.84 (overlapping signals with $^1J_{CP}$ couplings, PCH_2P), 61.97–63.76 (overlapping signals with $^2J_{CP}$ couplings, $POCH_2CH_3$), 72.51, 72.65, 72.84, 73.98, 74.05, 74.10, 74.32, 74.49, 75.03, 75.11, 75.14 (CH_2Ph), 76.11–78.84 (C-1, C-2, C-4, C-5 and C-6) 80.00, 80.11, 80.13 and 80.22 (C-3 in four diastereoisomers), 126.85–127.77 (Ph CH), 128.08–128.41 (Ph CH), 137.51, 137.57, 137.59, 137.63, 137.94, 138.04, 138.10, 138.21, 138.26, 138.28, 138.44, 138.49, 138.51, 138.54, 138.64 and 138.65 (16 signals, 4 × *ipso*-*C* of Ph in each of four diastereoisomers); ^{31}P NMR ($CDCl_3$, 162 MHz) δ 18.57–18.71 (overlapping d with $^2J_{PP}$ couplings), 18.99 (d, $^2J_{PP}$ 7.6 Hz), 19.32–19.48 (overlapping d with $^2J_{PP}$ couplings), 19.57 (d, $^2J_{PP}$ 5.0 Hz), 20.75 (d, $^2J_{PP}$ 4.2 Hz), 20.93 (d, $^2J_{PP}$ 4.5 Hz); HRMS (*m/z*) $[M+H]^+$ calcd. for $C_{48}H_{68}O_{16}P_4$; 1025.3531; found 1025.3553.

D-1,5-di-*O*-[[bis(ethyloxy)phosphorylmethyl]ethyloxyphosphoryl]-*myo*-inositol 2,3,4,6-tetrakis(dibenzylphosphate) (10)

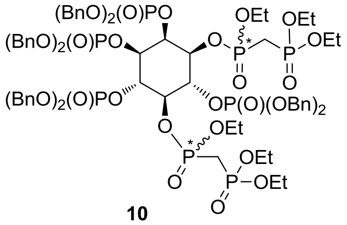

10

To a solution of **9** (74 mg, 0.072 mmol) in methanol (2 mL) and THF (2 mL) was added deionised water (0.5 mL), acetic acid (3 drops) and palladium hydroxide on activated charcoal (20%, 50% water, 40 mg). The suspension was stirred vigorously under an atmosphere of H_2 (balloon) for 2 days. TLC (ethyl acetate/methanol 2:1) showed that hydrogenolysis was complete with total conversion of **9** (R_f 0.80) into a more polar product (R_f 0.24, no UV activity, stain $KMnO_4$). The catalyst was removed by filtration through a PTFE syringe filter and the solution was concentrated and dried under vacuum to give crude tetraol as a colourless glass (44 mg). To a solution of this crude tetraol in dry dichloromethane (2.5 mL) was added 5-phenyltetrazole (58 mg, 0.40 mmol). The suspension was stirred under N_2 and bis(benzyloxy)diisopropylaminophosphine (0.12 mL, 0.35 mmol) was added. The mixture was stirred under N_2 at room temperature for 2 h and then cooled to –78 °C, before MCPBA (130 mg, 70%, 0.53 mmol) was added. The mixture was allowed to warm to room temperature and then diluted with EtOAc (20 mL). The clear, colourless solution was washed with 10% aq. Na_2SO_3 solution (3 × 20 mL), dried over $MgSO_4$ and concentrated. The residue was purified by flash chromatography (ethyl acetate, then methanol in ethyl acetate 0 to 20%) to give **10** as a colourless oil (79 mg, 0.046 mmole, 64% over two steps); ^{1}H NMR ($CDCl_3$, 400 MHz) δ 1.15–1.30 (18 H, m, 6 × OCH_2CH_3), 3.11 (4 H, m, 2 × PCH_2P), 3.98–4.33 (12 H, m, 6 × OCH_2CH_3), 4.43–5.23 (21 H, H-1, H-3, H-4, H-5, H-6 and 8 × $POCH_2Ph$), 5.50–5.54 (approx. 0.5 H, m, H-2 in two diastereosiomers), 5.63–5.66 (approx. 0.5 H, m, H-2 in two diastereosiomers), 7.13–7.38 (40, m, Ph); ^{13}C NMR ($CDCl_3$, 100 MHz) δ 16.09–16.36 (overlapping signals with $^3J_{CP}$ couplings, $POCH_2CH_3$), 25.51 (overlapping signals with $^1J_{CP}$ ~135 Hz, PCH_2P), 62.09–62.75 (overlapping signals with $^2J_{CP}$ couplings, $POCH_2CH_3$), 63.90–64.34 (overlapping signals with $^2J_{CP}$ couplings, $POCH_2CH_3$), 69.63–70.13 (overlapping signals with $^2J_{CP}$ couplings, $POCH_2Ph$), 72.04–76.32 (overlapping signals with J_{CP} couplings, inositol ring CH), 127.86–128.54 (CH of Ph), 135.64–135.97 (*ipso*-C of Ph); ^{31}P NMR ($CDCl_3$, 162 MHz) δ –2.55 to –0.47 (4 H, 15 lines, P-2, P-3, P-4 and P-6), 19.04–22.04 (4 H, 15 lines, 1-PCH_2P and 5-PCH_2P); HRMS (*m/z*) [M + Na]$^+$ calcd. for $C_{76}H_{96}O_{28}P_8$, 1727.3881; found 1727.3789.

D-*myo*-inositol 1,5-bis(methylenediphosphonate)-2,3,4,6-tetrakisphosphate (2)

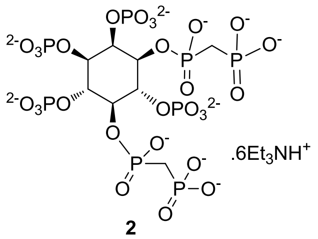

2

A stirred solution of **10** (40 mg, 23 µmole) in dry dichloromethane (1 mL) was cooled to 0 °C under N_2 and trimethylsilyl bromide (0.5 mL) was added dropwise over 5 min. The solution was allowed to warm gradually to room temperature, and stirring was continued for 60 h. The solution was concentrated and methanol (5 mL) was added to the residue. The resulting colourless solution was stirred at room temperature for a further 1 h, then concentrated to give a white gum. The gum was washed with diethyl ether (3 × 1 mL), then taken up in 1.0 moldm^{-3} TEAB (5 mL, pH 7.6) to give a cloudy suspension. This suspension was washed with diethyl ether (3 × 5 mL) to give a colourless, clear solution. This solution was concentrated under reduced pressure, re-dissolved in methanol and concentrated again. This cycle was repeated several times until a clear, colourless, glassy solid remained, which was finally re-dissolved in de-ionised water and lyophilised to give the triethylammonium salt of **2** as a brittle colourless solid (32 mg, 22 µmole, 96 %); $[\alpha]_D^{20}$ = +2.1 (*c* = 2.4, MeOH); ^{1}H NMR (D_2O, 400 MHz) δ 1.26 (~54 H, t, *J* 7.2 Hz, CH_3 of TEA^+), 2.30–2.49 (4 H, m, 2 × PCH_2P), 3.18 (~36 H, q, *J* 7.2 Hz, CH_2 of TEA^+), 4.25 (1 H, broad t, *J* ~9.8 Hz, H-3), 4.34–4.57 (4 H, m, H-1, H-4, H-5 and H-6), approx. 4.8 (1 H, buried by HDO, H-2); ^{13}C NMR (D_2O, 100 MHz) δ 8.21 (CH_3 of TEA^+), 28.30 (two overlapping t, $^1J_{CP}$ ~ 130 Hz, 2 × PCH_2P), 71.80, 73.31, 75.20, 75.58, and 75.80 (5 × inositol ring *C*H), 76.04 (C-2); ^{31}P NMR (D_2O, 162 MHz) δ –0.80 (1 P, phosphate P), –0.13 (1 P, phosphate P), 0.24 (1 P, phosphate P), 0.58 (1 P, phosphate P), 14.36 (1 P, $^2J_{PP}$ 10.8 Hz, phosphonate P), 14.60 (1 P, $^2J_{PP}$ 10.5 Hz, phosphonate P), 19.97 (1 P, $^2J_{PP}$ 10.4 Hz, phosphonate P), 20.32 (1 P, $^2J_{PP}$ 10.8 Hz, phosphonate P); HRMS (*m/z*) $[M]^-$ calcd. for $C_8H_{24}O_{28}P_8$, 814.8277; found 814.8317.

The ^{31}P NMR spectrum of this material (see SI) showed a signal at δ 15.88, probably originating from a minor impurity in some batches of **8**. This impurity could be removed from **2** by ion-exchange by chromatography on Q-Sepharose Fast Flow resin, eluting with a gradient of 0 to 2.0 moldm^{-3} TEAB. The additional purification step reduced the yield of **2** to 74% from **10**.

NMR Spectra

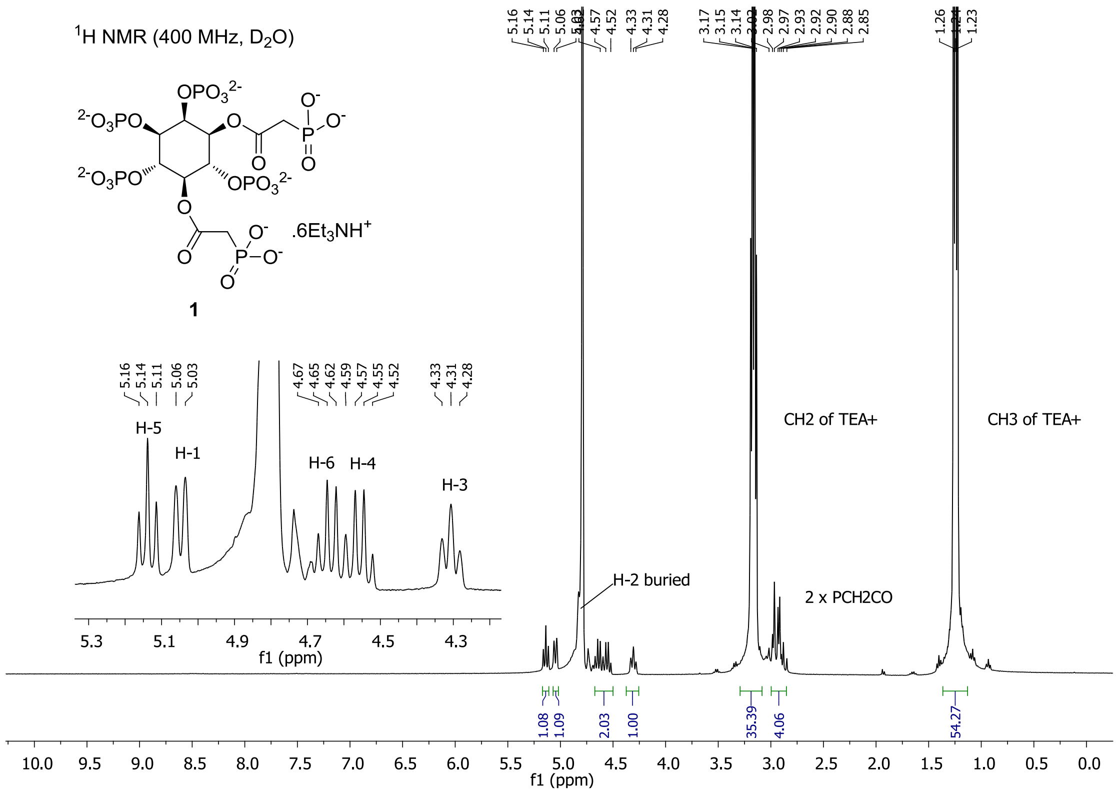

¹H NMR (400 MHz, D₂O)
.6Et₃NH⁺
1
5.16 5.14 5.11 5.06 5.03
4.67 4.65 4.62 4.59 4.57 4.55 4.52
4.33 4.31 4.28
3.17 3.15 3.14 2.98 2.97 2.93 2.92 2.90 2.88 2.85
1.26 1.23
H-5
H-1
H-6
H-4
H-3
CH2 of TEA+
CH3 of TEA+
H-2 buried
2 x PCH2CO
1.08 1.09 2.03 1.00 35.39 4.06 54.27
f1 (ppm)

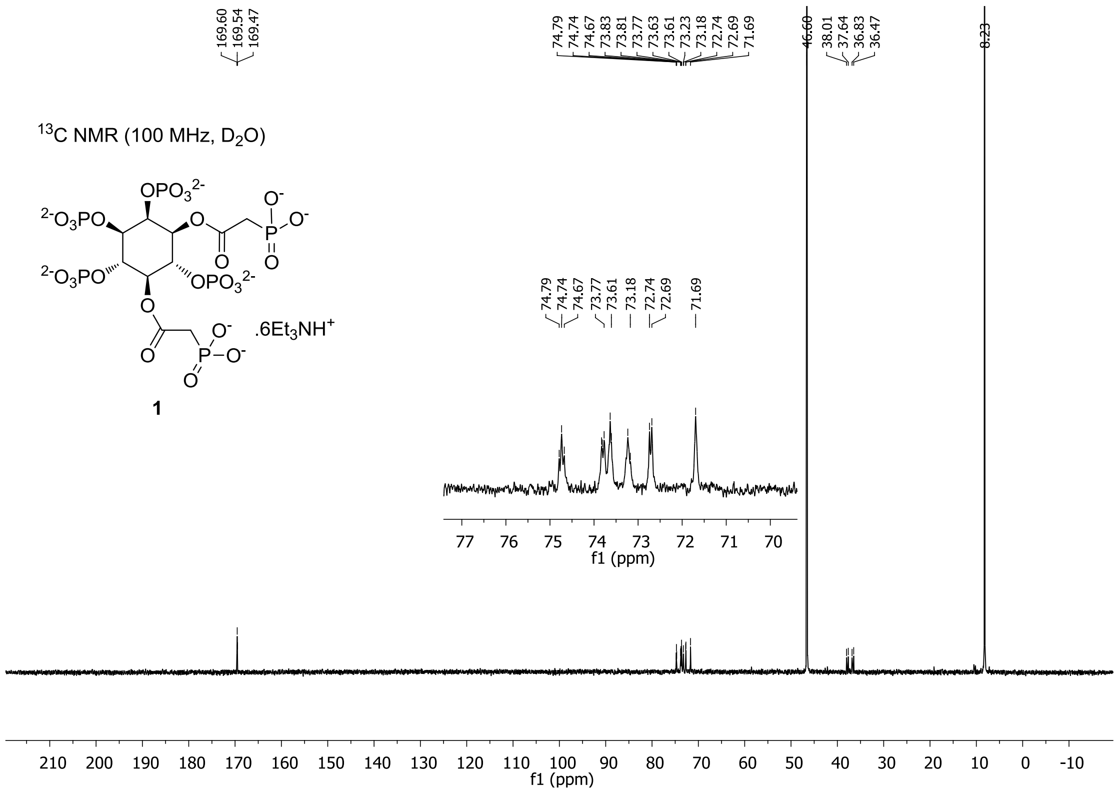

169.60 169.54 169.47
74.79 74.74 74.67 73.83 73.81 73.77 73.63 73.61 73.23 73.18 72.74 72.69 71.69
46.60 38.01 37.64 36.83 36.47 8.23
¹³C NMR (100 MHz, D₂O)
.6Et₃NH⁺
1
74.79 74.74 74.67 73.77 73.61 73.18 72.74 72.69 71.69
f1 (ppm)

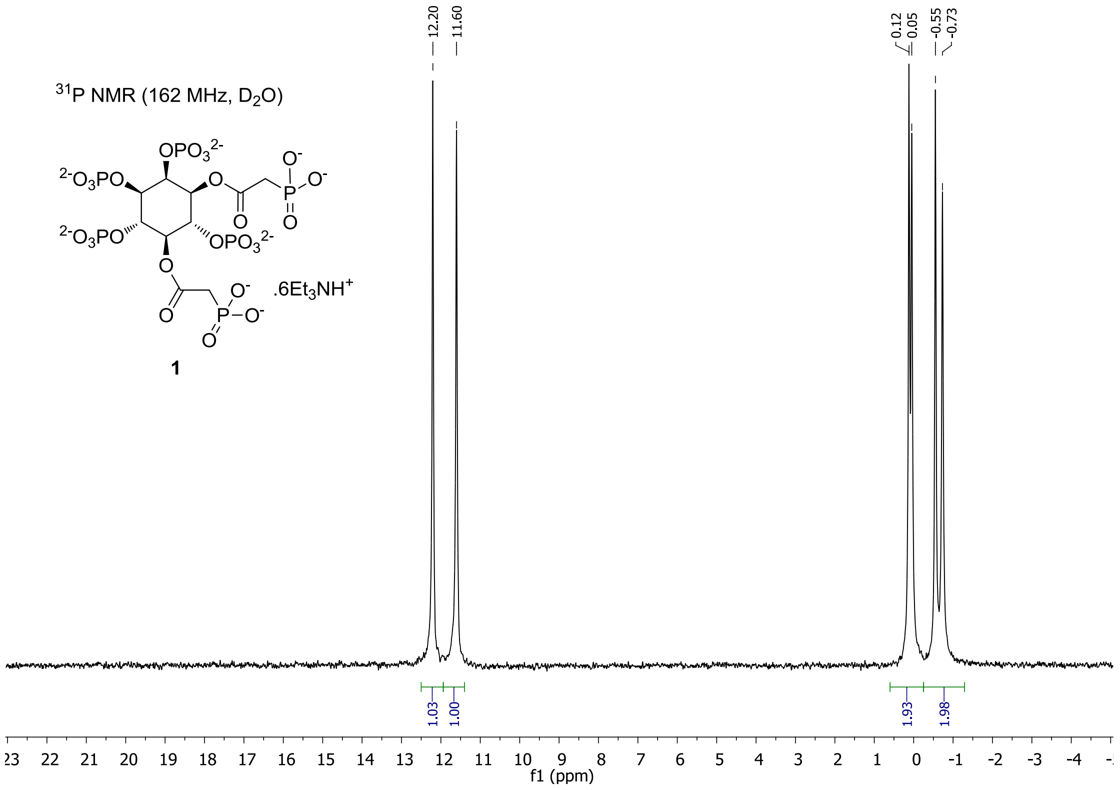

31P NMR (162 MHz, D2O)
.6Et3NH+
1
12.20
11.60
0.12
0.05
-0.55
-0.73
1.03
1.00
1.93
1.98
f1 (ppm)

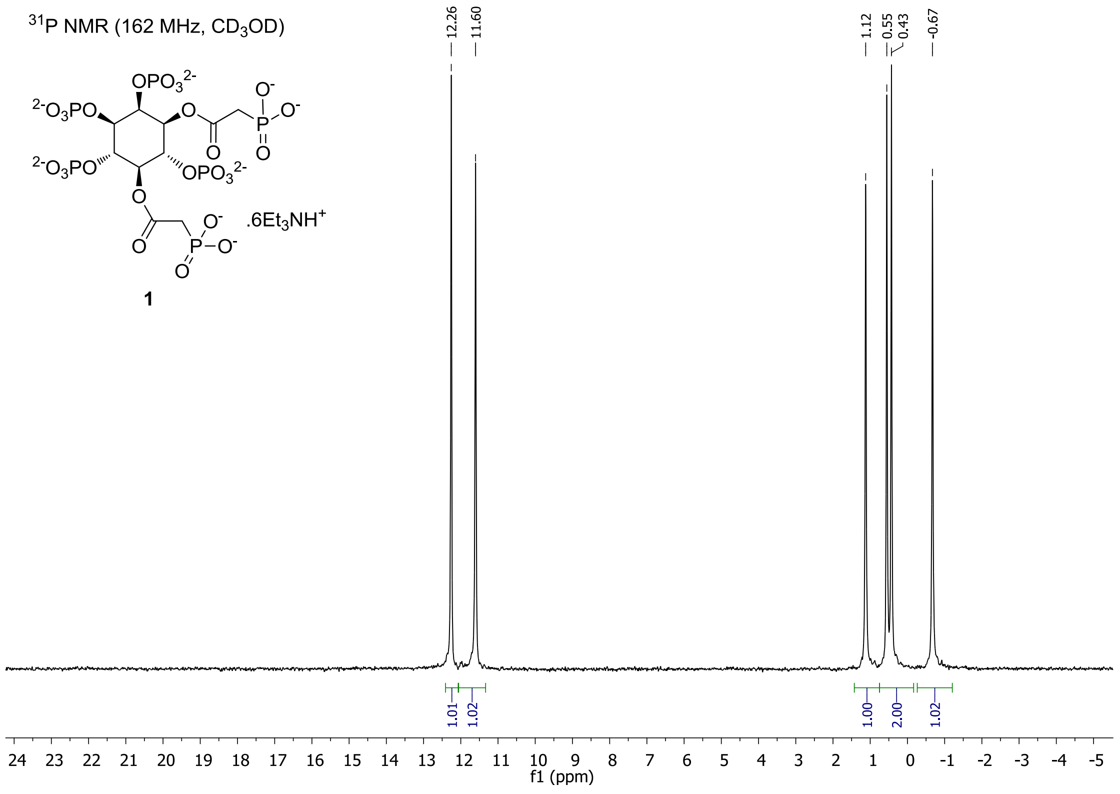

31P NMR (162 MHz, CD3OD)
.6Et3NH+
1
12.26
11.60
1.12
0.55
0.43
-0.67
1.01
1.02
1.00
2.00
1.02
f1 (ppm)

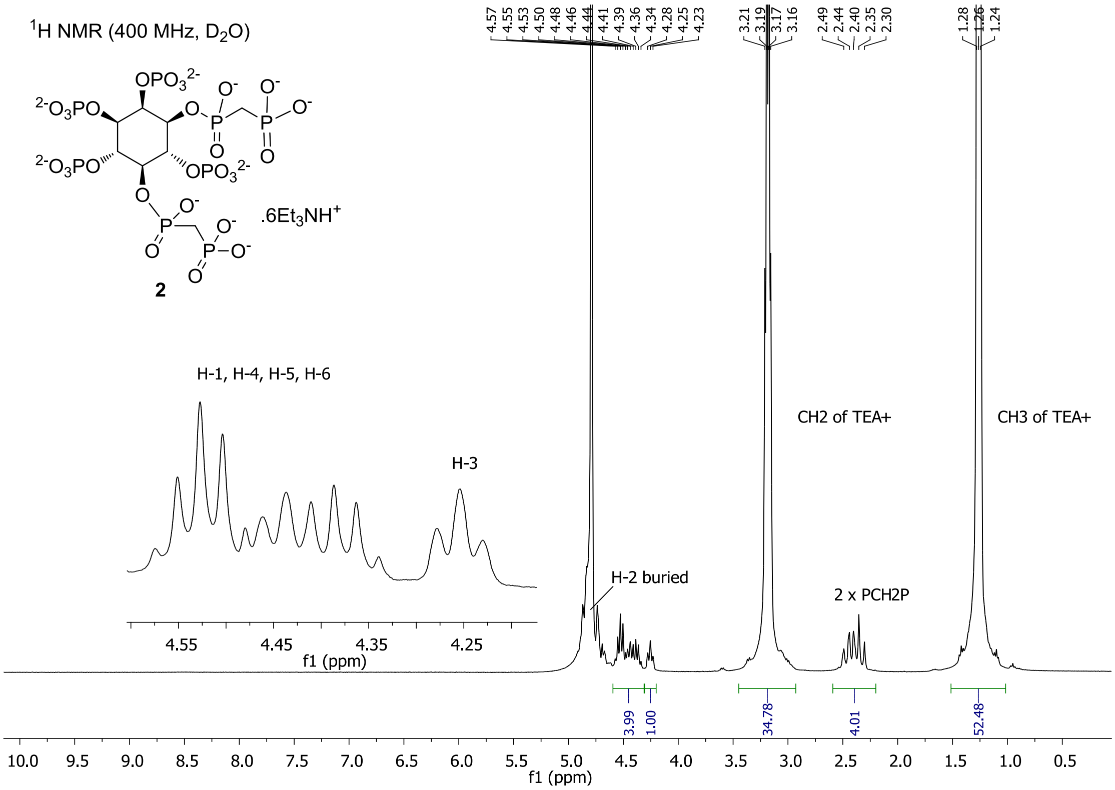

1H NMR (400 MHz, D2O)
.6Et3NH+
2
H-1, H-4, H-5, H-6
H-3
H-2 buried
CH2 of TEA+
CH3 of TEA+
2 x PCH2P
4.57 4.55 4.53 4.50 4.48 4.46 4.44 4.41 4.39 4.36 4.34 4.28 4.25 4.23
3.21 3.19 3.17 3.16
2.49 2.44 2.40 2.35 2.30
1.28 1.26 1.24
3.99 1.00 34.78 4.01 52.48
f1 (ppm)

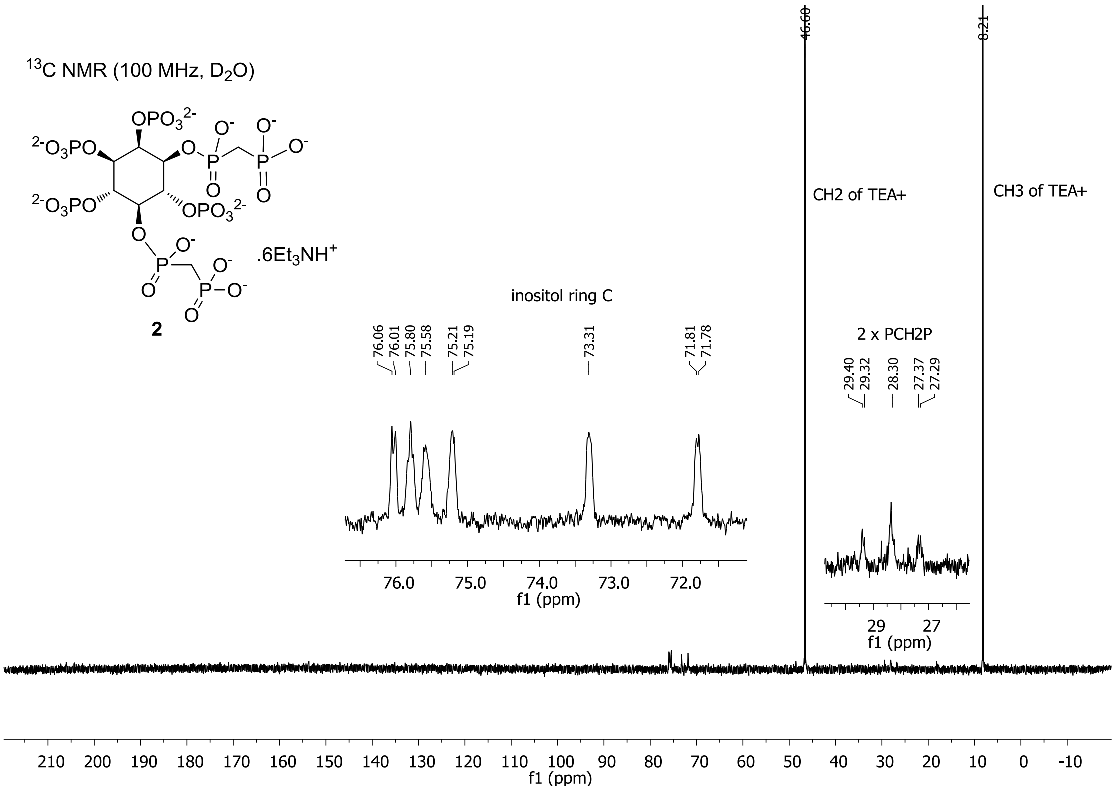

13C NMR (100 MHz, D2O)
.6Et3NH+
2
inositol ring C
CH2 of TEA+
CH3 of TEA+
2 x PCH2P
76.06 76.01 75.80 75.58 75.21 75.19
73.31
71.81 71.78
29.40 29.32 28.30 27.37 27.29
46.60
8.21
f1 (ppm)

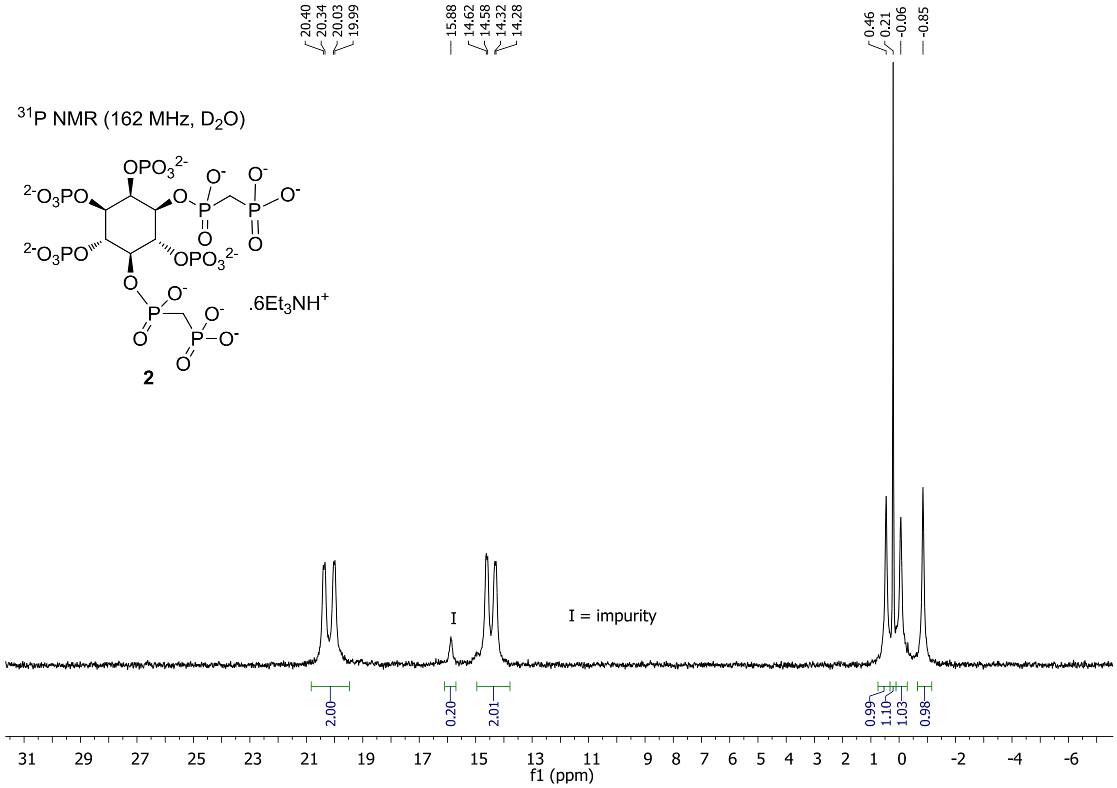

20.40
20.34
20.03
19.99
15.88
14.62
14.58
14.32
14.28
0.46
0.21
-0.06
-0.85
31P NMR (162 MHz, D2O)
.6Et3NH+
2
I
I = impurity
2.00
0.20
2.01
0.99
1.10
1.03
0.98
f1 (ppm)

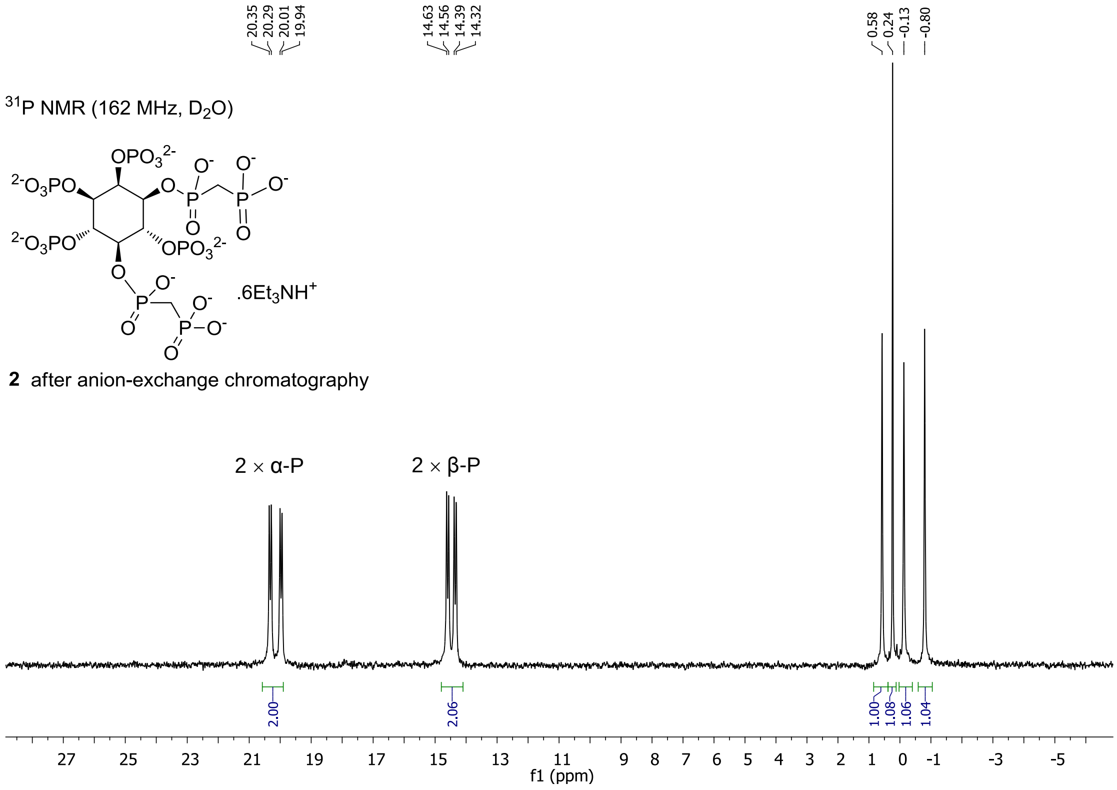

20.35
20.29
20.01
19.94
14.63
14.56
14.39
14.32
0.58
0.24
-0.13
-0.80
31P NMR (162 MHz, D2O)
.6Et3NH+
2 after anion-exchange chromatography
2 × α-P
2 × β-P
2.00
2.06
1.00
1.08
1.06
1.04
f1 (ppm)

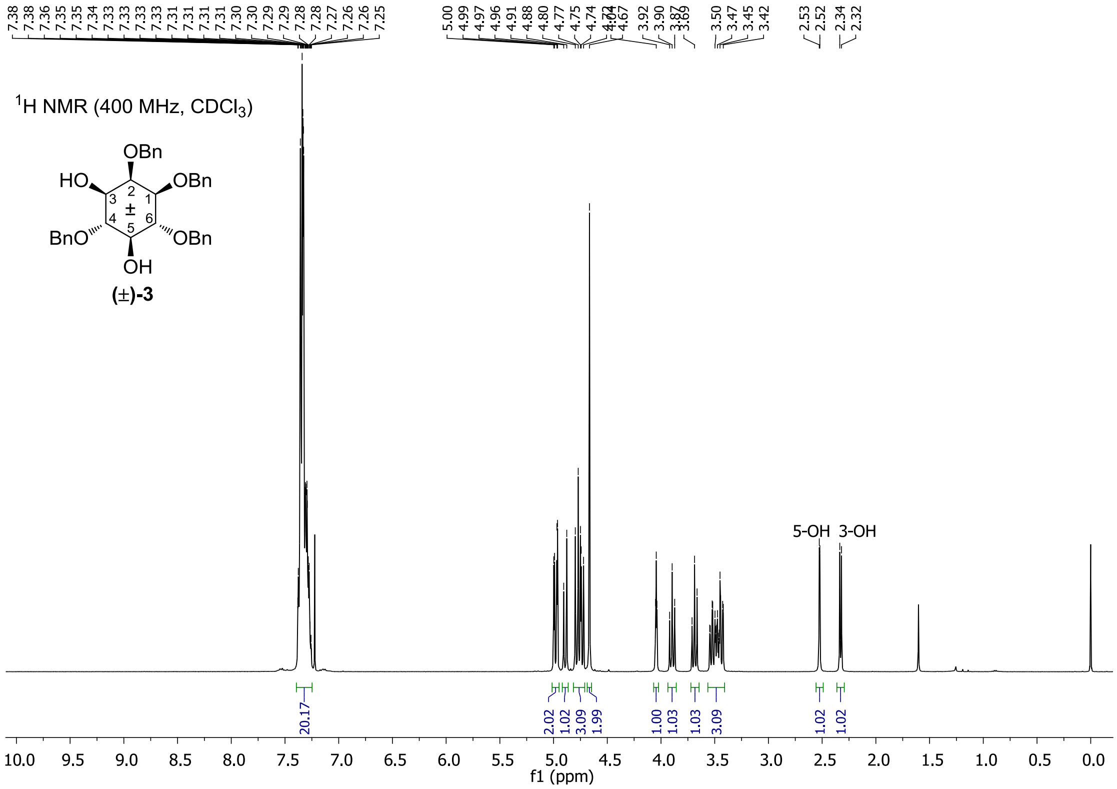
7.38 7.38 7.36 7.35 7.35 7.34 7.33 7.33 7.33 7.33 7.31 7.31 7.31 7.30 7.30 7.29 7.29 7.28 7.28 7.27 7.26 7.26 7.25
5.00 4.99 4.97 4.96 4.91 4.88 4.80 4.77 4.75 4.74 4.67
3.92 3.90 3.87 3.69 3.50 3.47 3.45 3.42
2.53 2.52 2.34 2.32
1H NMR (400 MHz, CDCl3)
OBn
HO
OBn
BnO
OBn
OH
(±)-3
5-OH 3-OH
20.17
2.02 1.02 3.09 1.99
1.00 1.03 1.03 3.09
1.02 1.02
10.0 9.5 9.0 8.5 8.0 7.5 7.0 6.5 6.0 5.5 5.0 4.5 4.0 3.5 3.0 2.5 2.0 1.5 1.0 0.5 0.0
f1 (ppm)

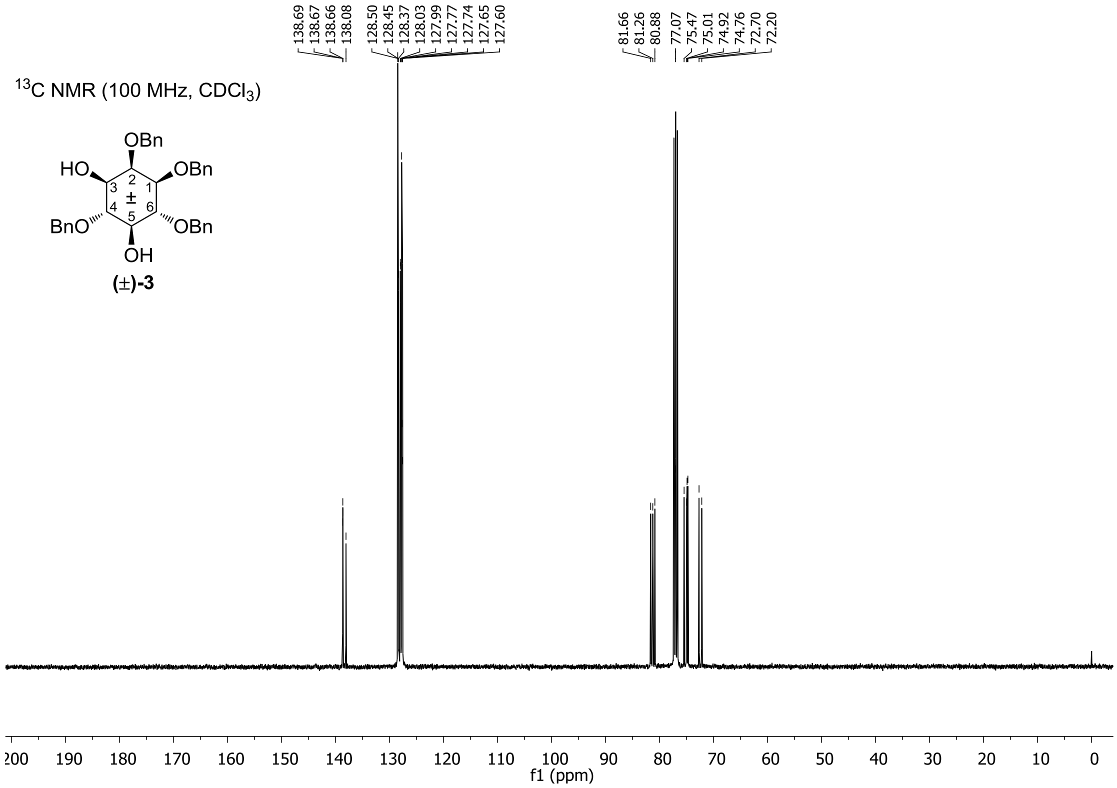
138.69 138.67 138.66 138.08
128.50 128.45 128.37 128.03 127.99 127.77 127.74 127.65 127.60
81.66 81.26 80.88 77.07 75.47 75.01 74.92 74.76 72.70 72.20
13C NMR (100 MHz, CDCl3)
OBn
HO
OBn
BnO
OBn
OH
(±)-3
200 190 180 170 160 150 140 130 120 110 100 90 80 70 60 50 40 30 20 10 0
f1 (ppm)

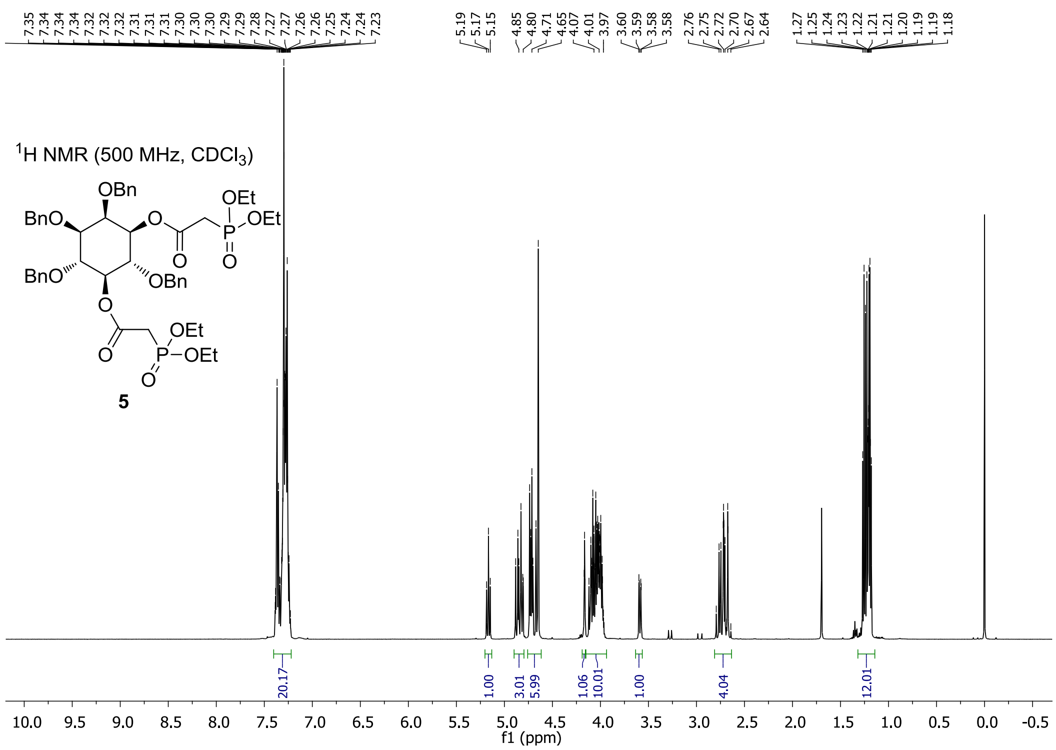

^{1}H NMR (500 MHz, CDCl$_3$)
5
f1 (ppm)

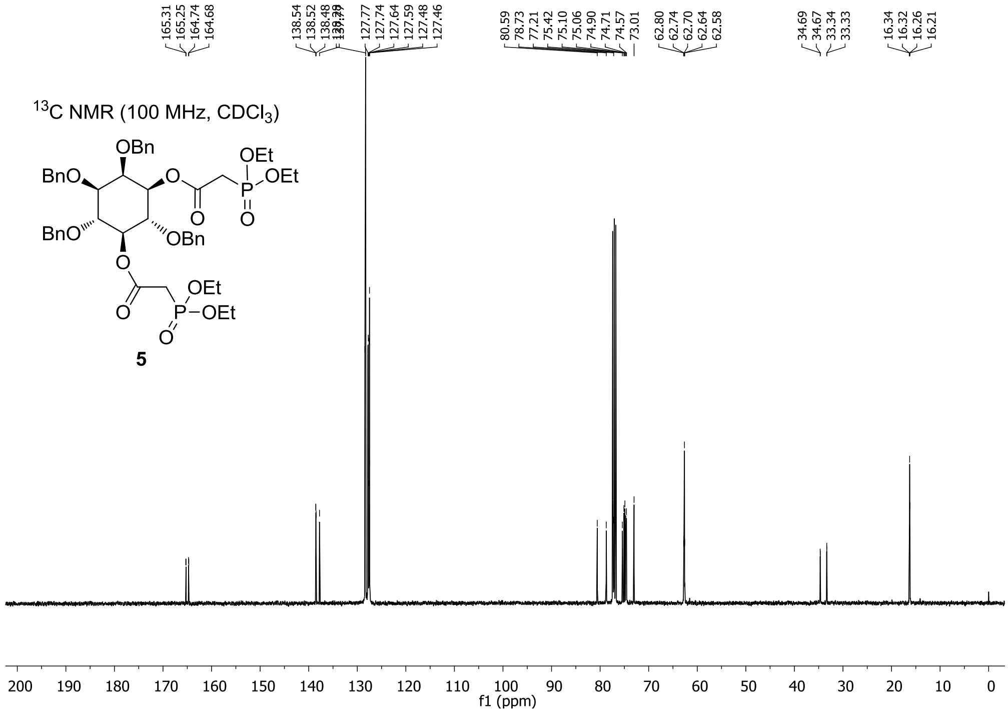

^{13}C NMR (100 MHz, CDCl$_3$)
5
f1 (ppm)

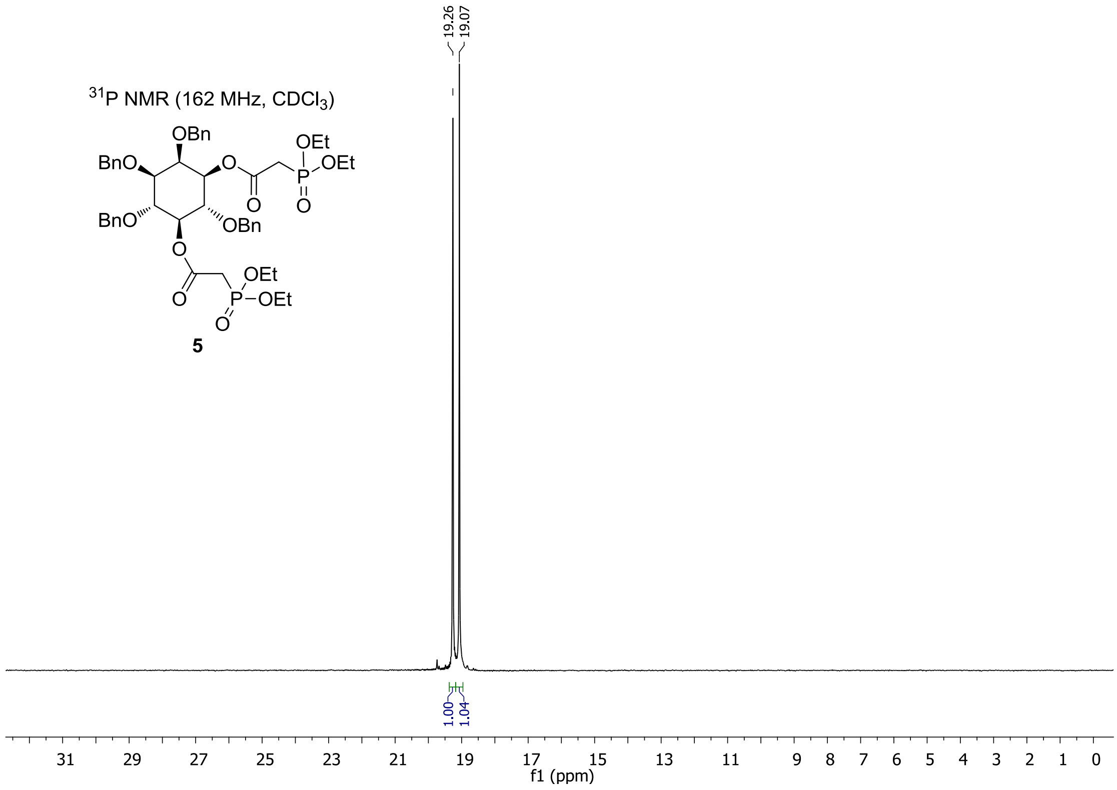
31P NMR (162 MHz, CDCl3)
OBn
BnO
BnO
OBn
OEt
OEt
P
O
O
O
OEt
P-OEt
5
19.26
19.07
1.00
1.04
f1 (ppm)

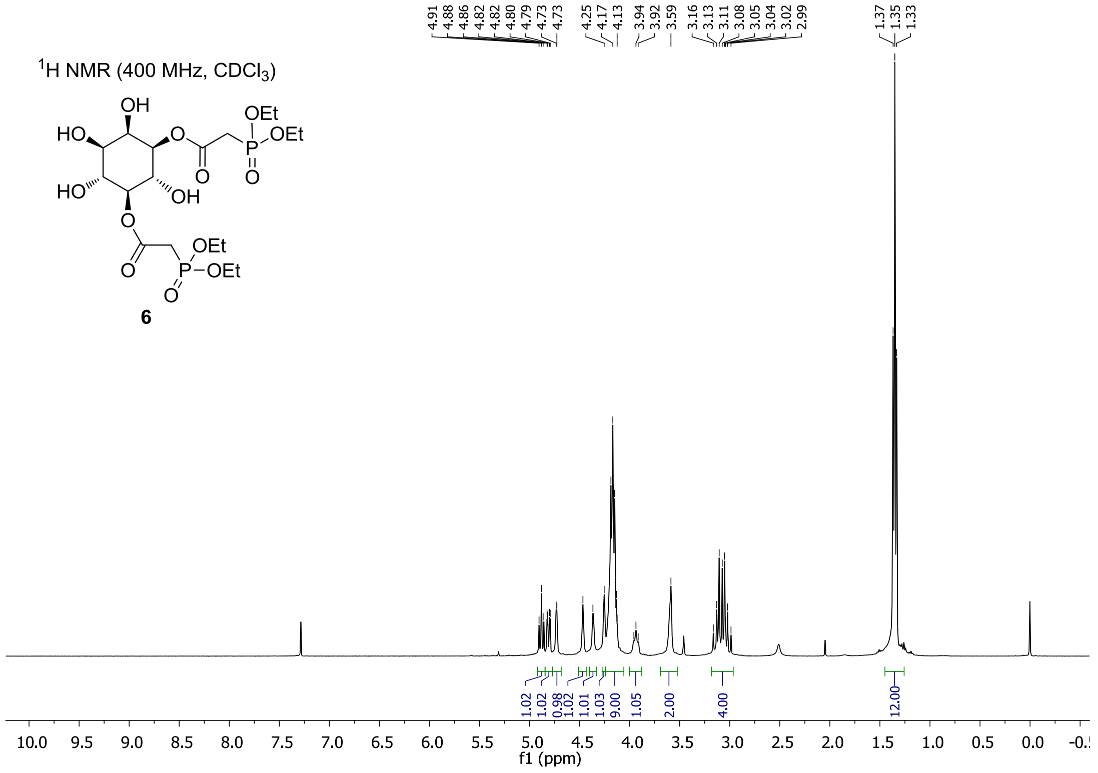
1H NMR (400 MHz, CDCl3)
OH
HO
HO
OH
OEt
OEt
P
O
O
O
OEt
P-OEt
6
4.91 4.88 4.86 4.82 4.82 4.80 4.79 4.73 4.73
4.25 4.17 4.13 3.94 3.92 3.59
3.16 3.13 3.11 3.08 3.05 3.04 3.02 2.99
1.37 1.35 1.33
1.02 1.02 0.98 1.02 1.01 1.03 9.00 1.05 2.00 4.00 12.00
f1 (ppm)

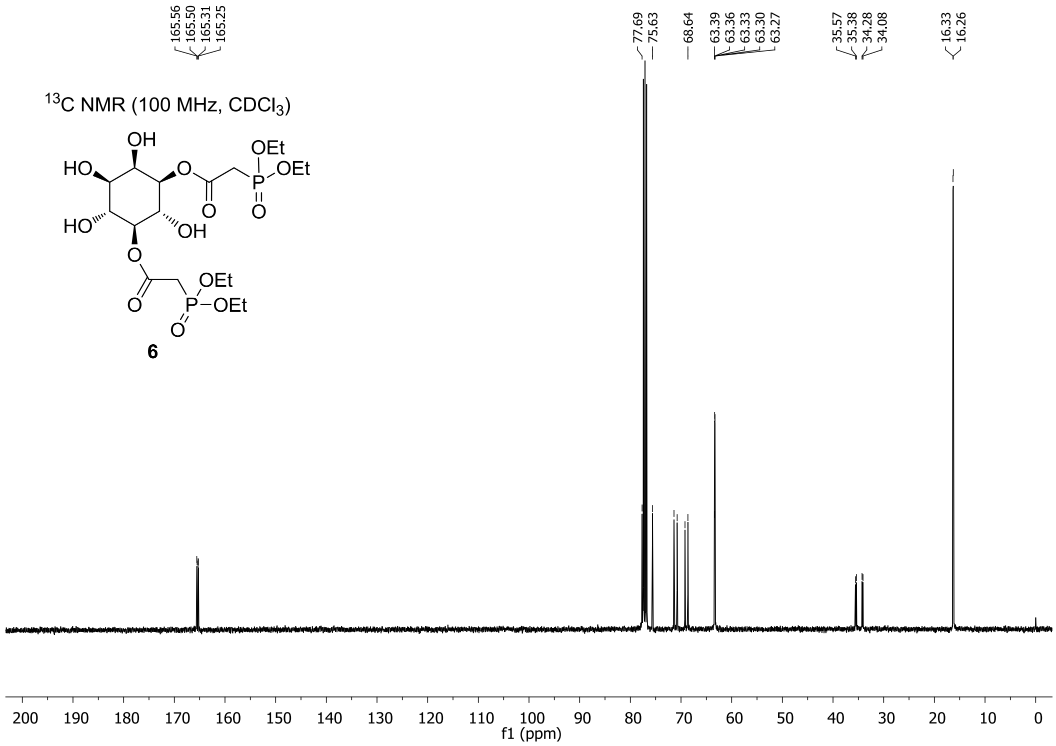
165.56
165.50
165.31
165.25
77.69
75.63
68.64
63.39
63.36
63.33
63.30
63.27
35.57
35.38
34.28
34.08
16.33
16.26
^{13}C NMR (100 MHz, CDCl$_3$)
OH
HO
O
OEt
P
OEt
O
O
HO
OH
O
O
OEt
P
OEt
O
6
200 190 180 170 160 150 140 130 120 110 100 90 80 70 60 50 40 30 20 10 0
f1 (ppm)

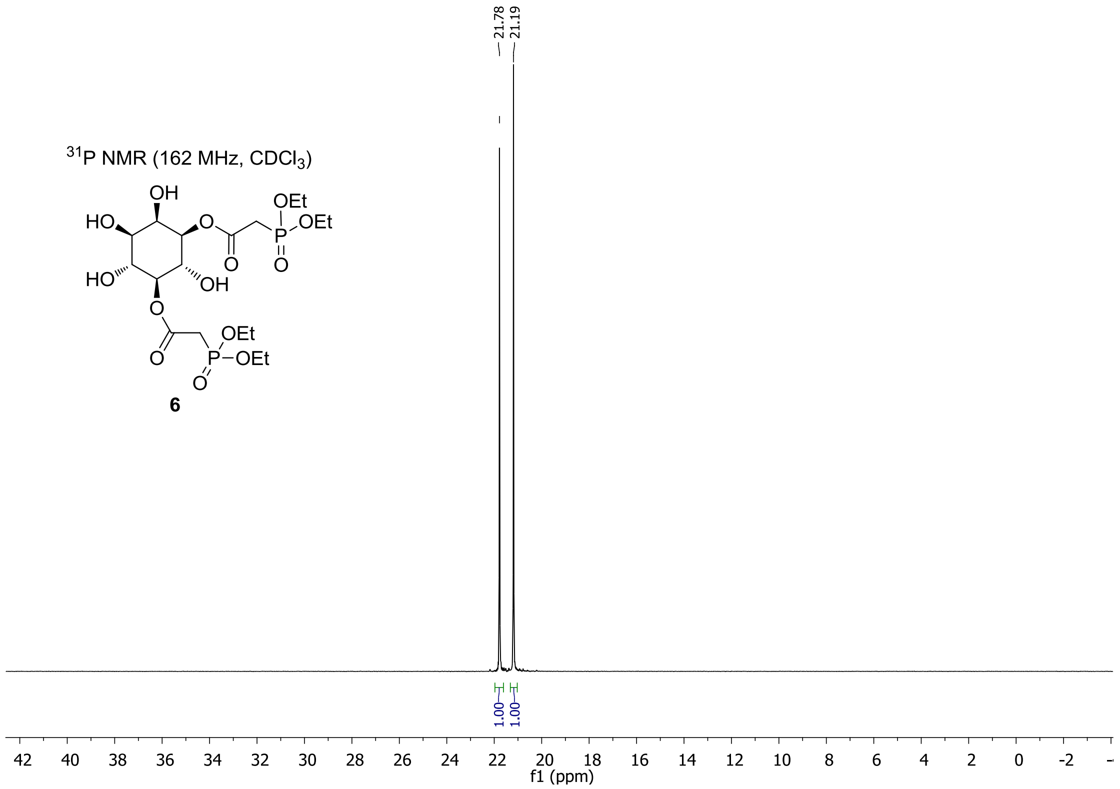
21.78
21.19
^{31}P NMR (162 MHz, CDCl$_3$)
OH
HO
O
OEt
P
OEt
O
O
HO
OH
O
O
OEt
P
OEt
O
6
1.00
1.00
42 40 38 36 34 32 30 28 26 24 22 20 18 16 14 12 10 8 6 4 2 0 -2
f1 (ppm)

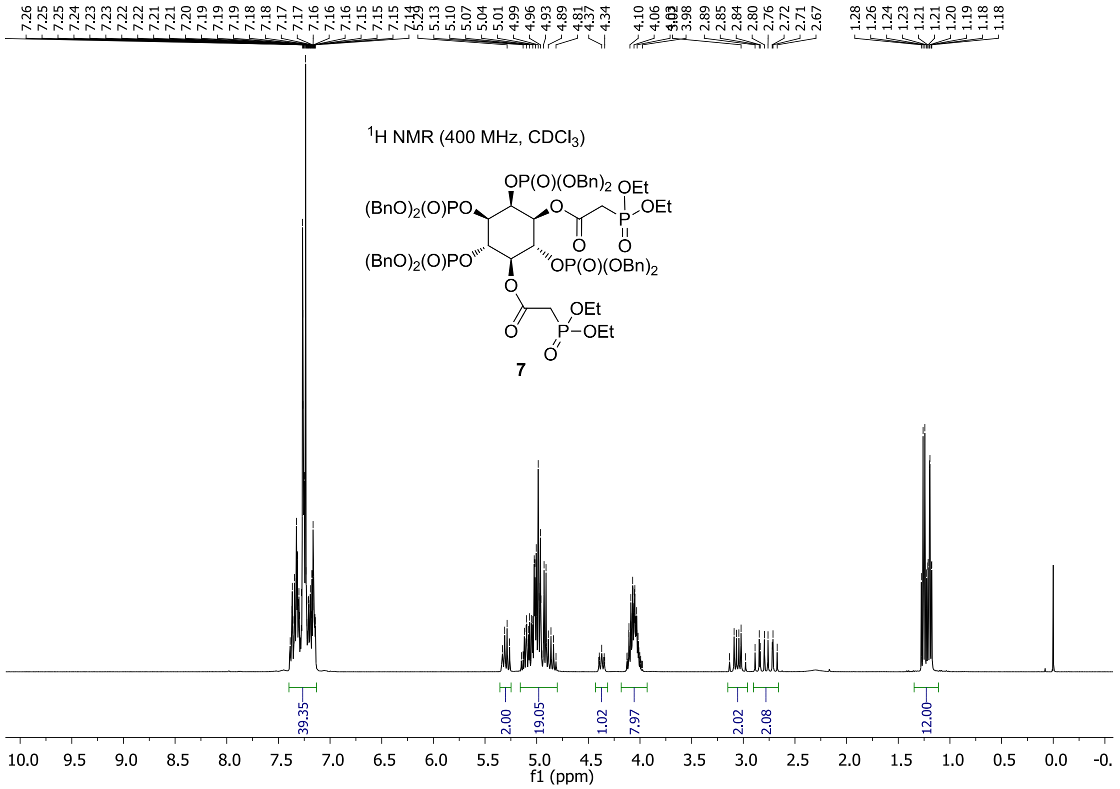
1H NMR (400 MHz, CDCl3)
7
f1 (ppm)

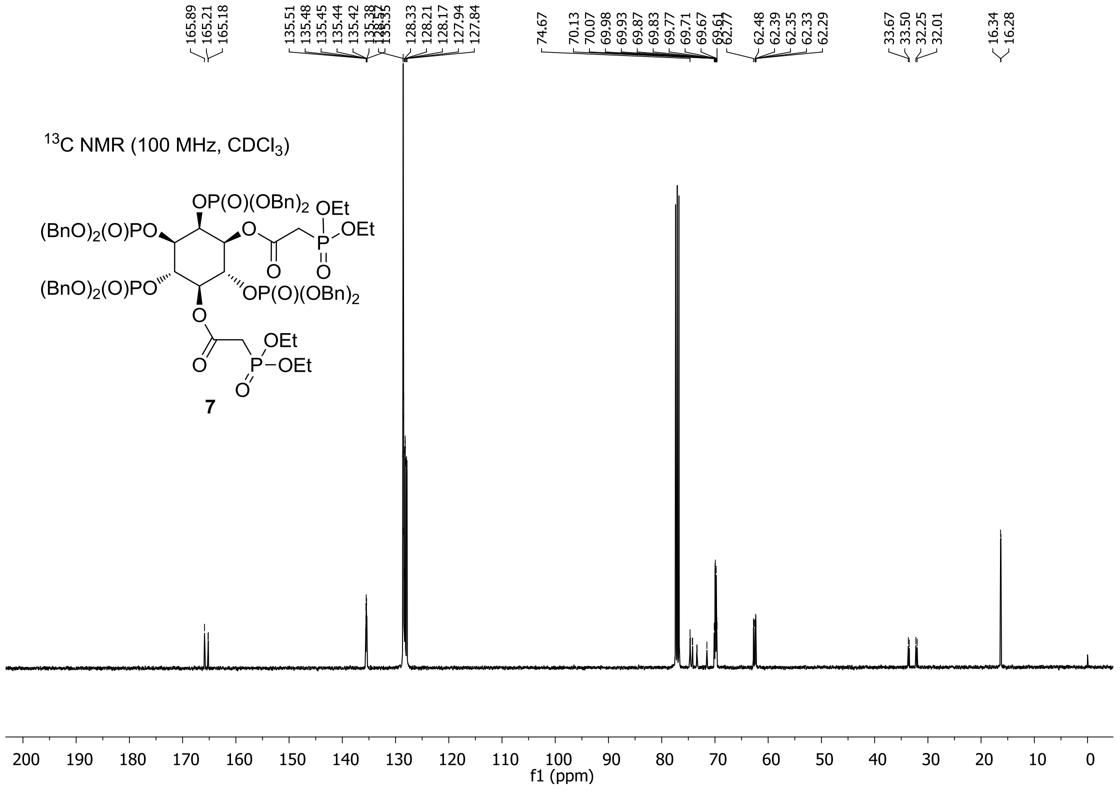
13C NMR (100 MHz, CDCl3)
7
f1 (ppm)

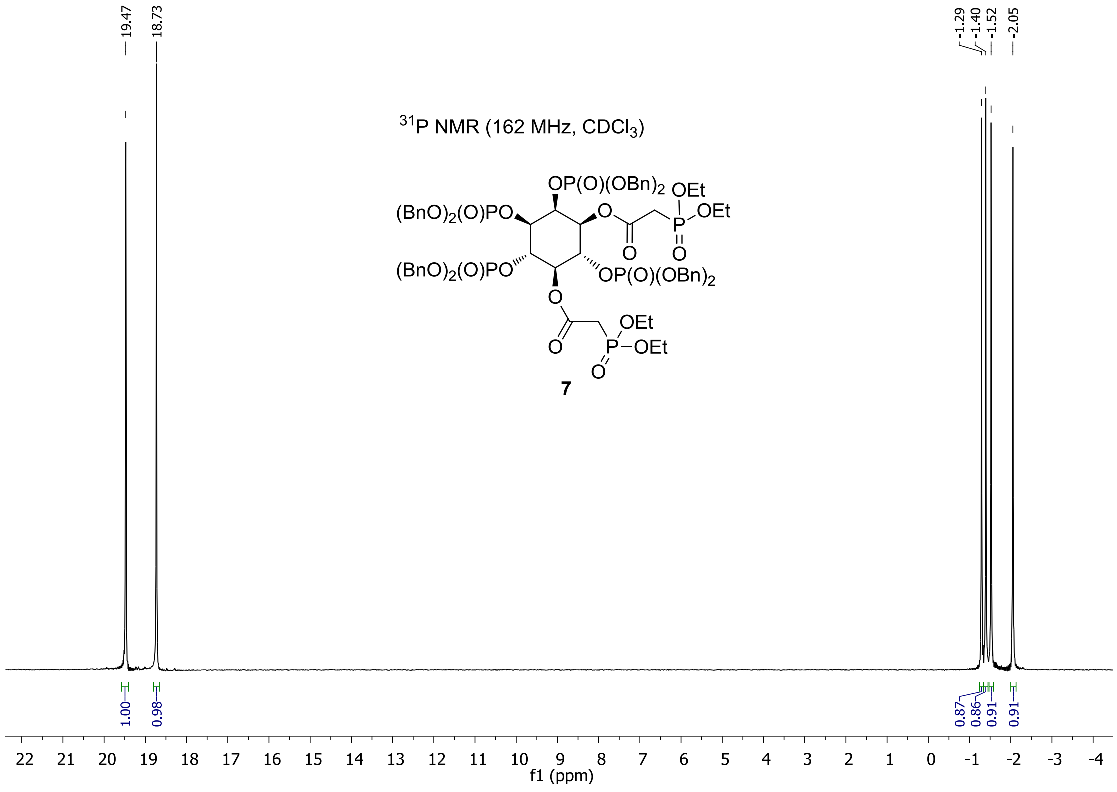

19.47
18.73
-1.29
-1.40
-1.52
-2.05
31P NMR (162 MHz, CDCl3)
OP(O)(OBn)2
OEt
(BnO)2(O)PO
O
P
OEt
O
O
(BnO)2(O)PO
OP(O)(OBn)2
O
OEt
O
P
OEt
O
7
1.00
0.98
0.87
0.86
0.91
0.91
22 21 20 19 18 17 16 15 14 13 12 11 10 9 8 7 6 5 4 3 2 1 0 -1 -2 -3 -4
f1 (ppm)

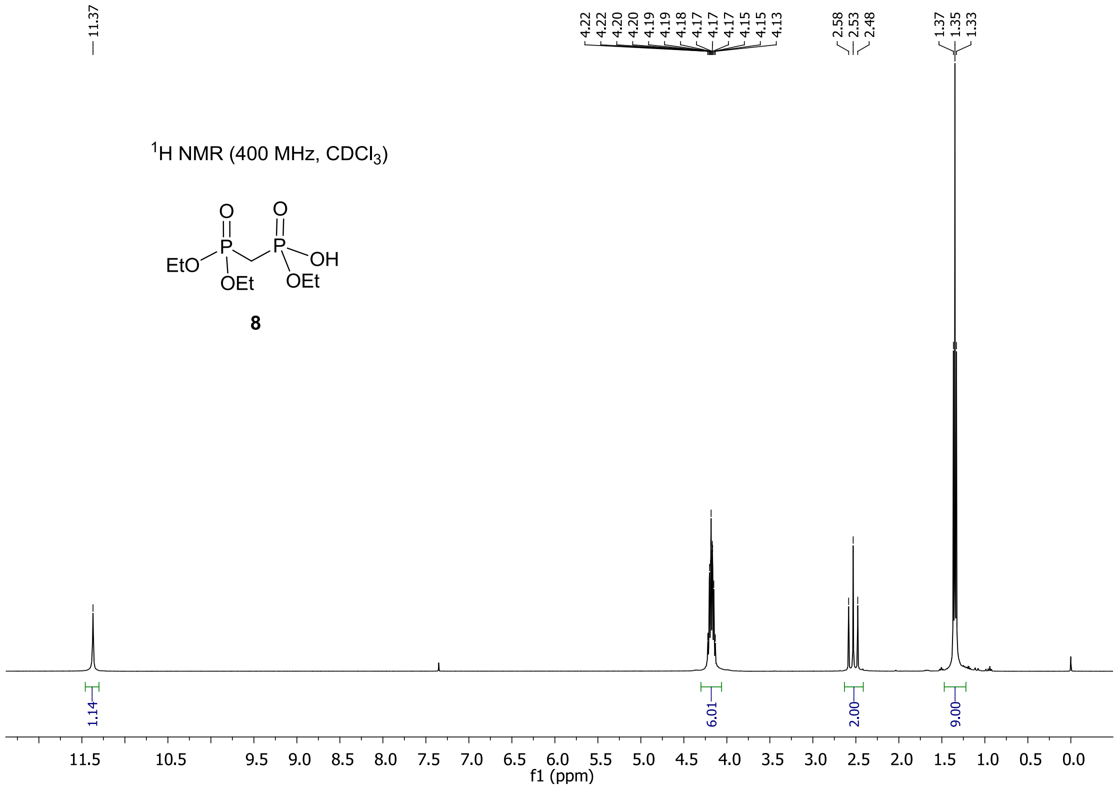

11.37
4.22
4.22
4.20
4.20
4.19
4.19
4.18
4.17
4.17
4.17
4.15
4.15
4.13
2.58
2.53
2.48
1.37
1.35
1.33
1H NMR (400 MHz, CDCl3)
O
O
P
P
EtO
OH
OEt
OEt
8
1.14
6.01
2.00
9.00
11.5 10.5 9.5 9.0 8.5 8.0 7.5 7.0 6.5 6.0 5.5 5.0 4.5 4.0 3.5 3.0 2.5 2.0 1.5 1.0 0.5 0.0
f1 (ppm)

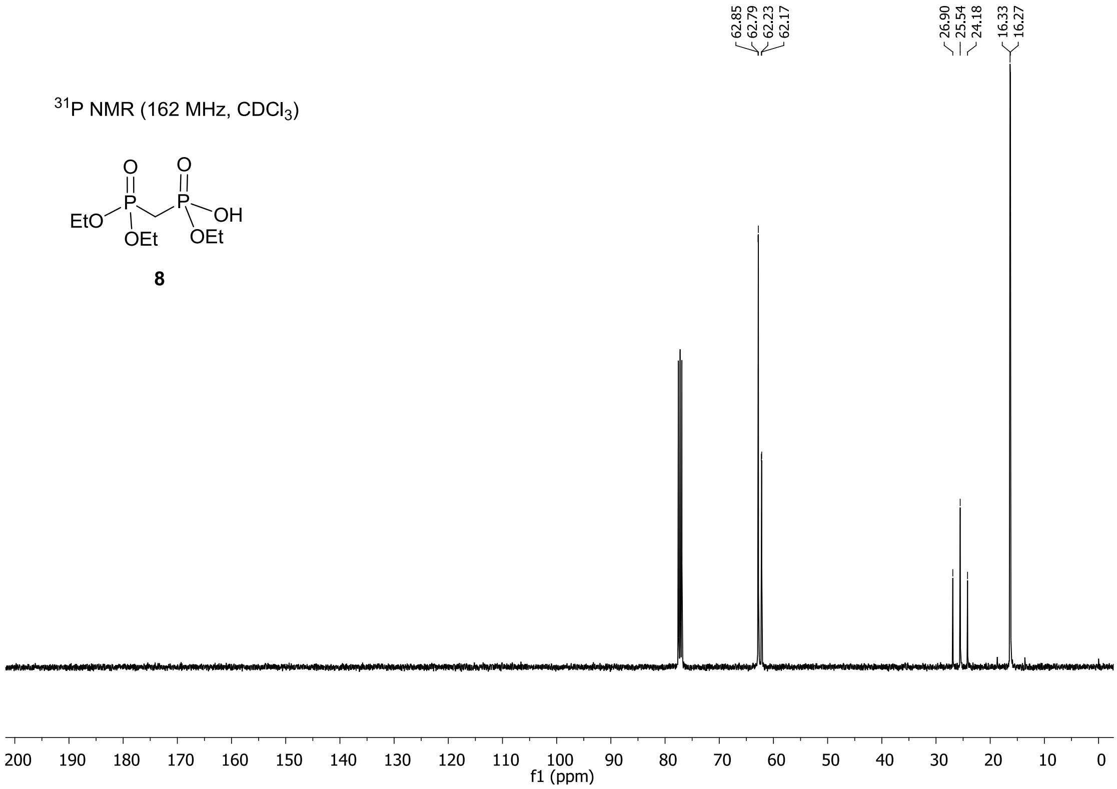

^{31}P NMR (162 MHz, $CDCl_3$)
8
62.85
62.79
62.23
62.17
26.90
25.54
24.18
16.33
16.27
200 190 180 170 160 150 140 130 120 110 100 90 80 70 60 50 40 30 20 10 0
f1 (ppm)

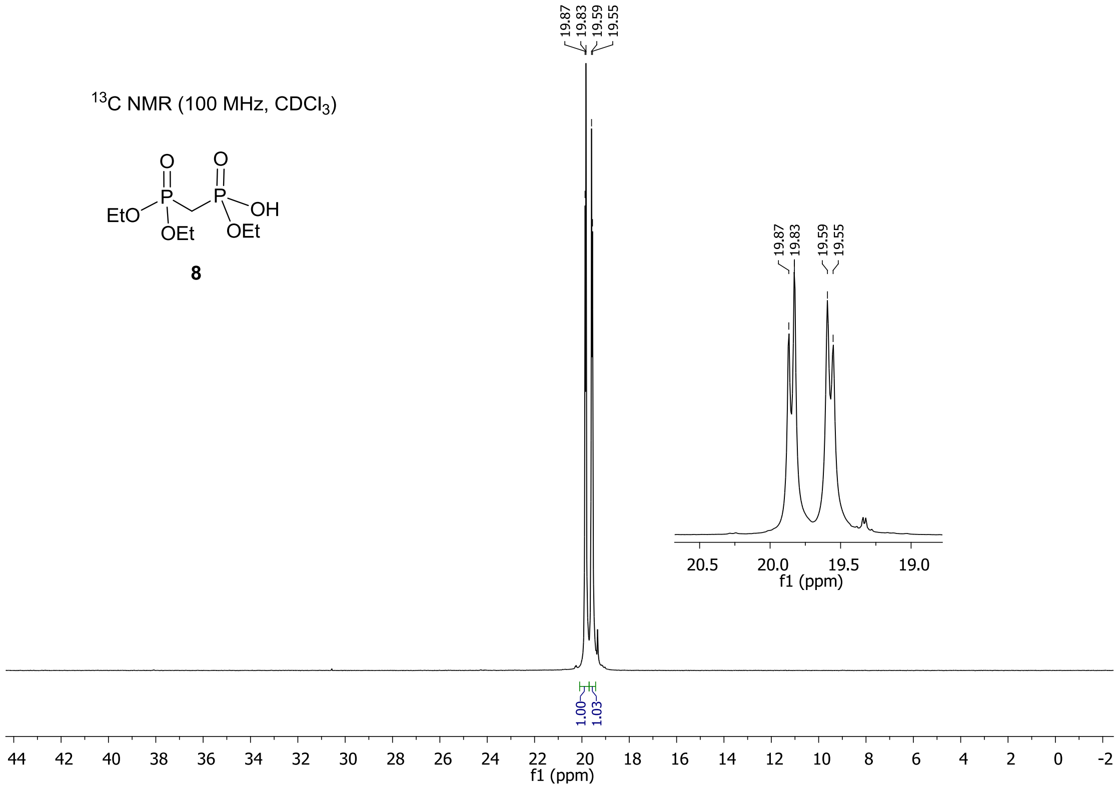

^{13}C NMR (100 MHz, $CDCl_3$)
8
19.87
19.83
19.59
19.55
1.00
1.03
20.5 20.0 19.5 19.0
f1 (ppm)
44 42 40 38 36 34 32 30 28 26 24 22 20 18 16 14 12 10 8 6 4 2 0 -2
f1 (ppm)

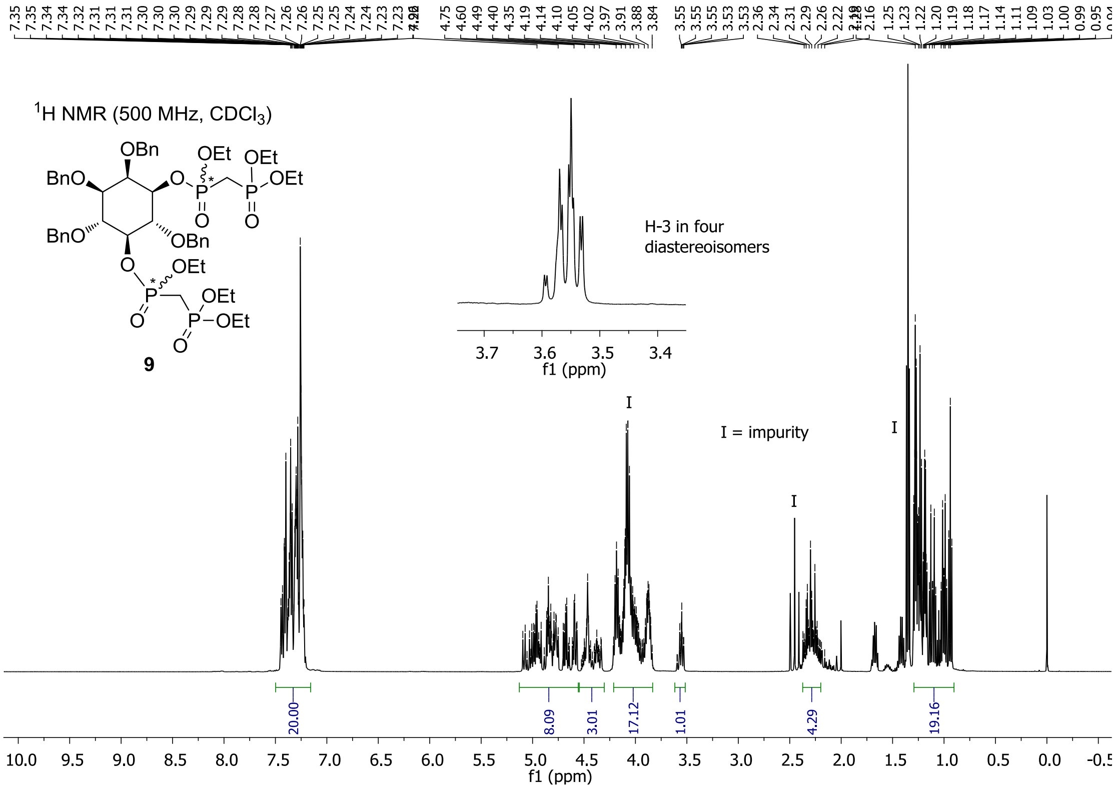

1H NMR (500 MHz, CDCl3)
9
H-3 in four diastereoisomers
I = impurity
f1 (ppm)

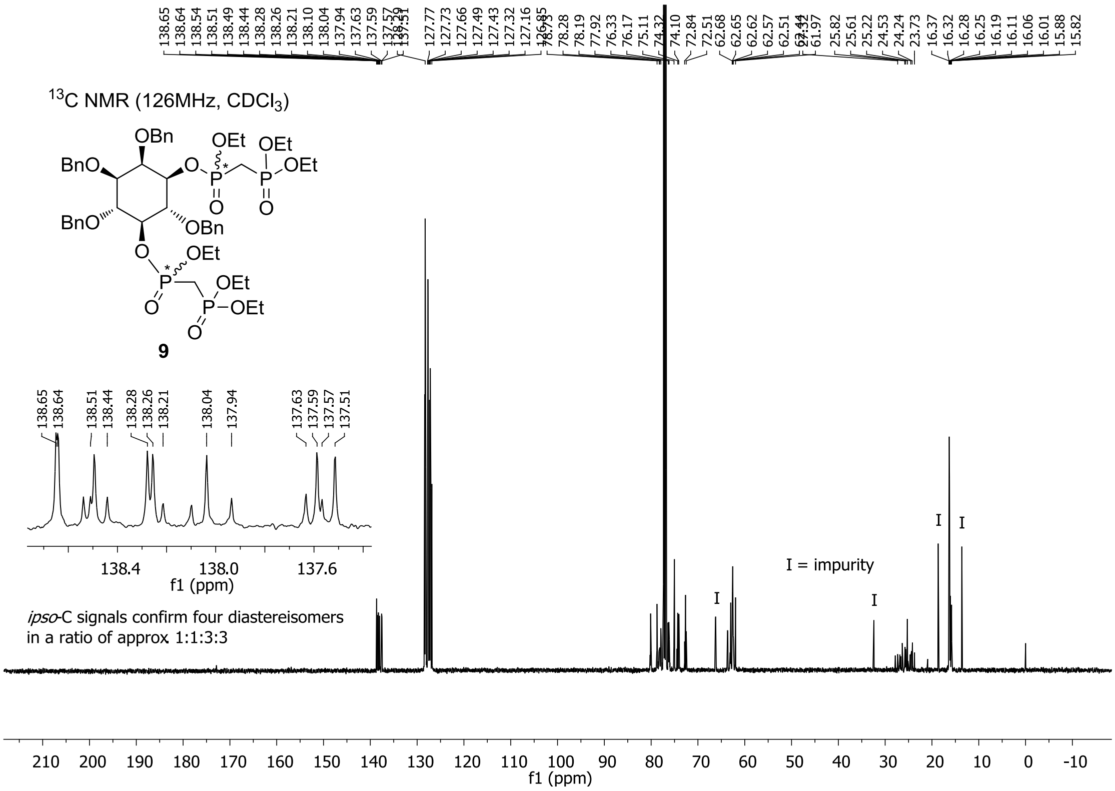

13C NMR (126MHz, CDCl3)
9
ipso-C signals confirm four diastereoisomers in a ratio of approx 1:1:3:3
I = impurity
f1 (ppm)

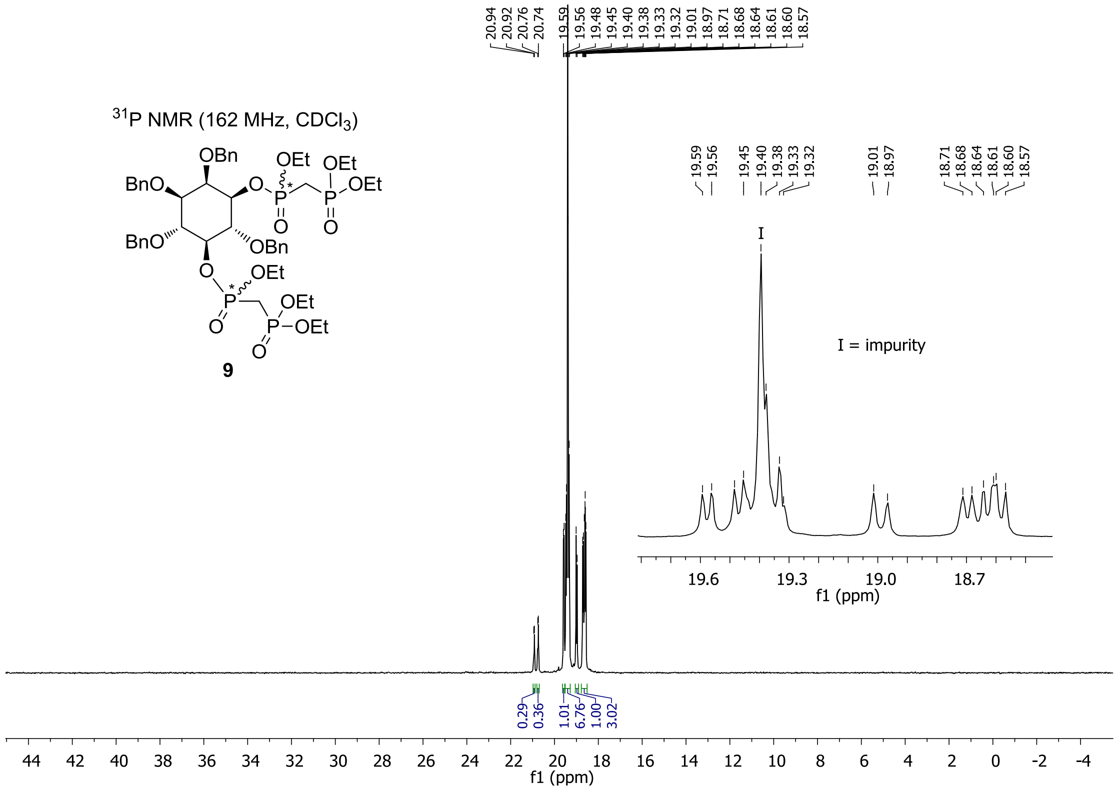

31P NMR (162 MHz, CDCl3)
9
I = impurity
f1 (ppm)

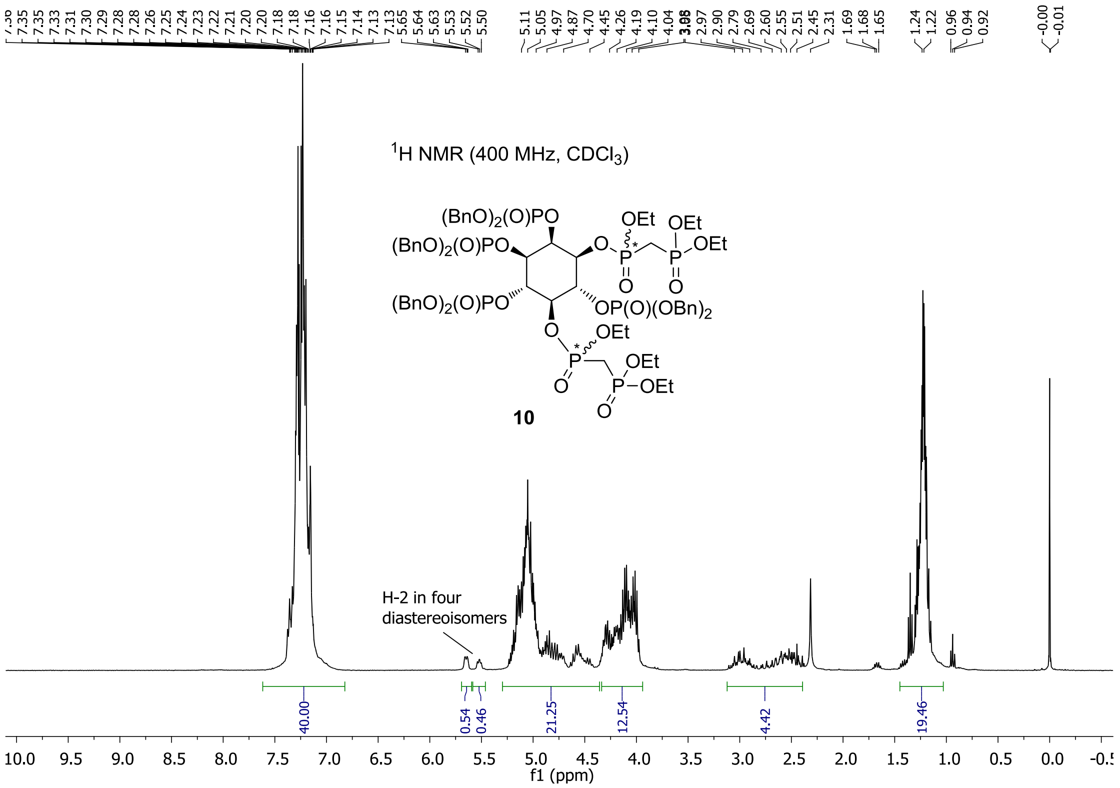

1H NMR (400 MHz, CDCl3)
10
H-2 in four diastereoisomers
f1 (ppm)

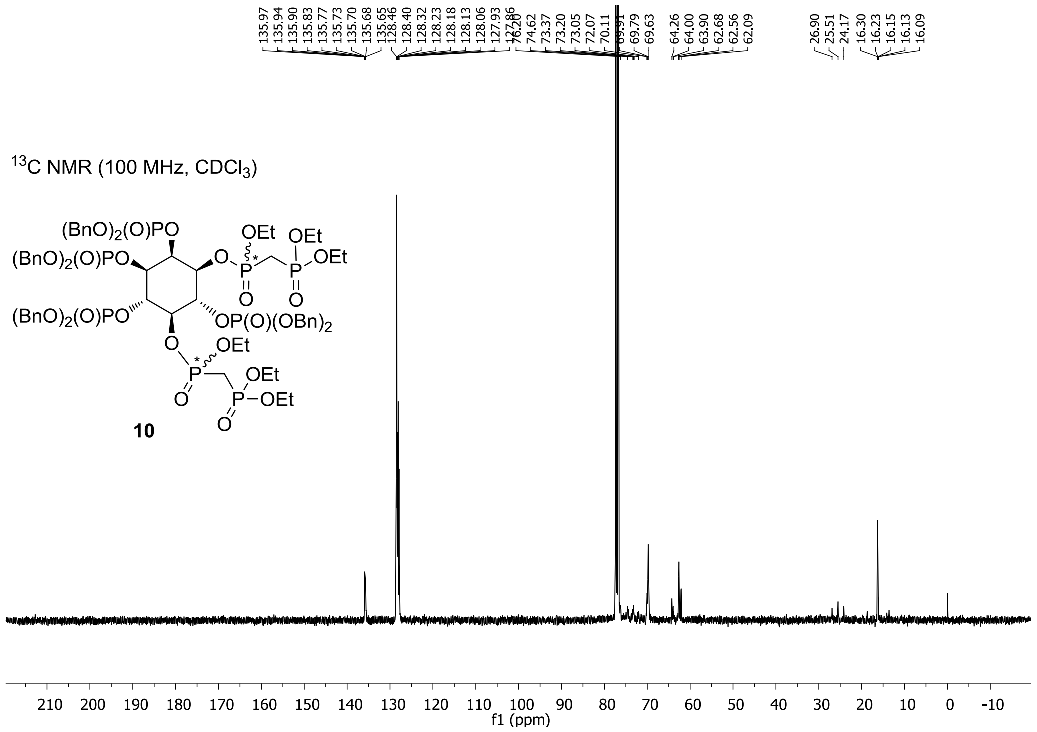
135.97
135.94
135.90
135.83
135.77
135.73
135.70
135.68
135.65
128.46
128.40
128.32
128.23
128.18
128.13
128.06
127.93
74.62
73.37
73.20
73.05
72.07
70.11
69.79
69.63
64.26
64.00
63.90
62.68
62.56
62.09
26.90
25.51
24.17
16.30
16.23
16.15
16.13
16.09
13C NMR (100 MHz, CDCl3)
(BnO)2(O)PO
(BnO)2(O)PO
(BnO)2(O)PO
OP(O)(OBn)2
OEt
OEt
OEt
OEt
OEt
OEt
10
f1 (ppm)

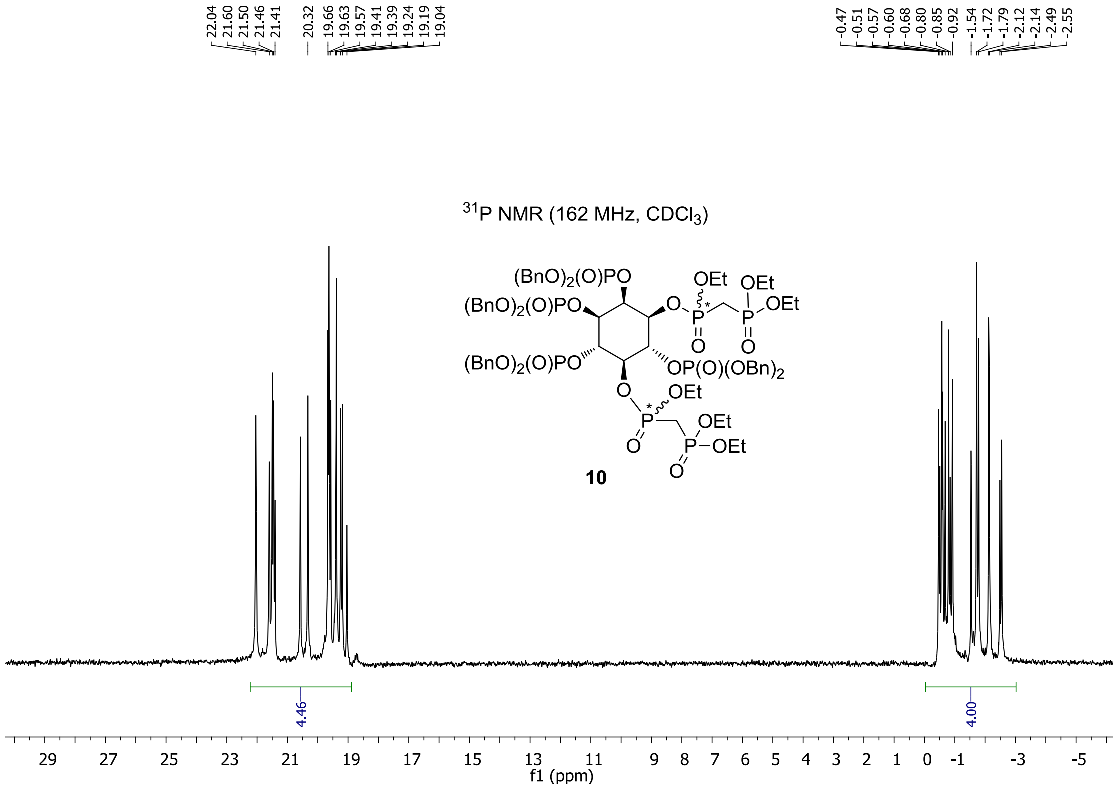
22.04
21.60
21.50
21.46
21.41
20.32
19.66
19.63
19.57
19.41
19.39
19.24
19.19
19.04
-0.47
-0.51
-0.57
-0.60
-0.68
-0.80
-0.85
-0.92
-1.54
-1.72
-1.79
-2.12
-2.14
-2.49
-2.55
31P NMR (162 MHz, CDCl3)
(BnO)2(O)PO
(BnO)2(O)PO
(BnO)2(O)PO
OP(O)(OBn)2
OEt
OEt
OEt
OEt
OEt
OEt
10
4.46
4.00
f1 (ppm)

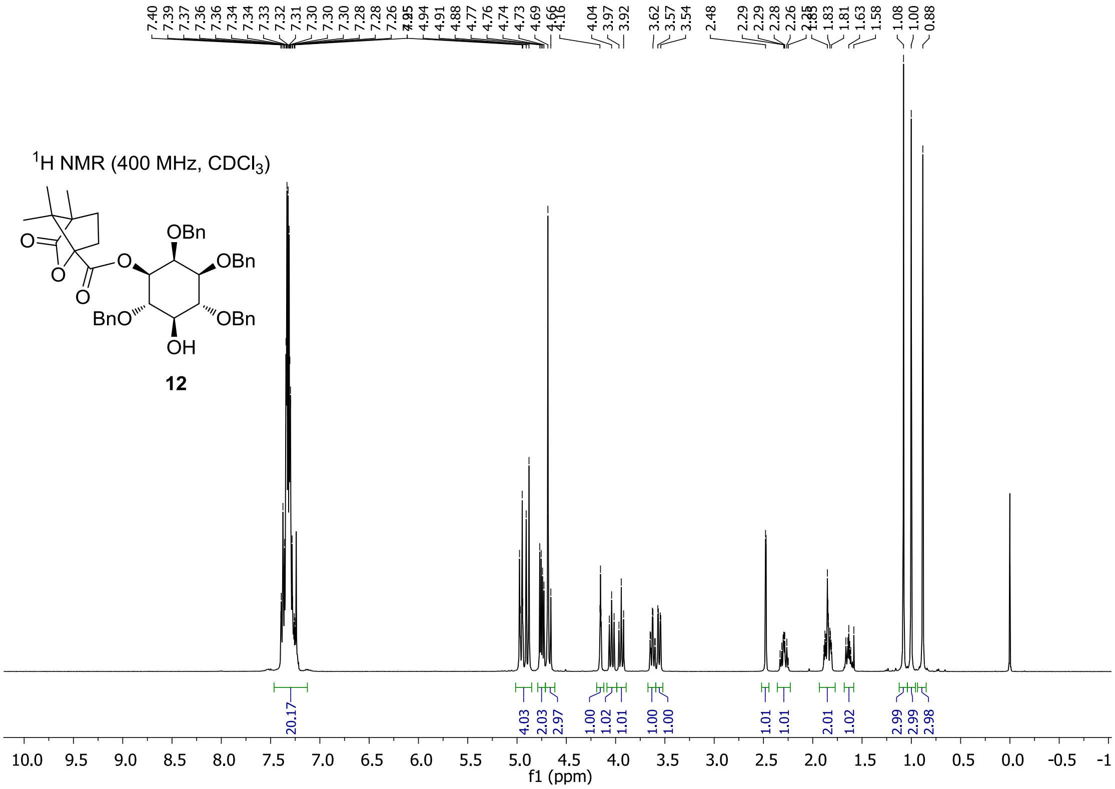

1H NMR (400 MHz, CDCl3)
OBn
OBn
BnO
OBn
OH
12
f1 (ppm)

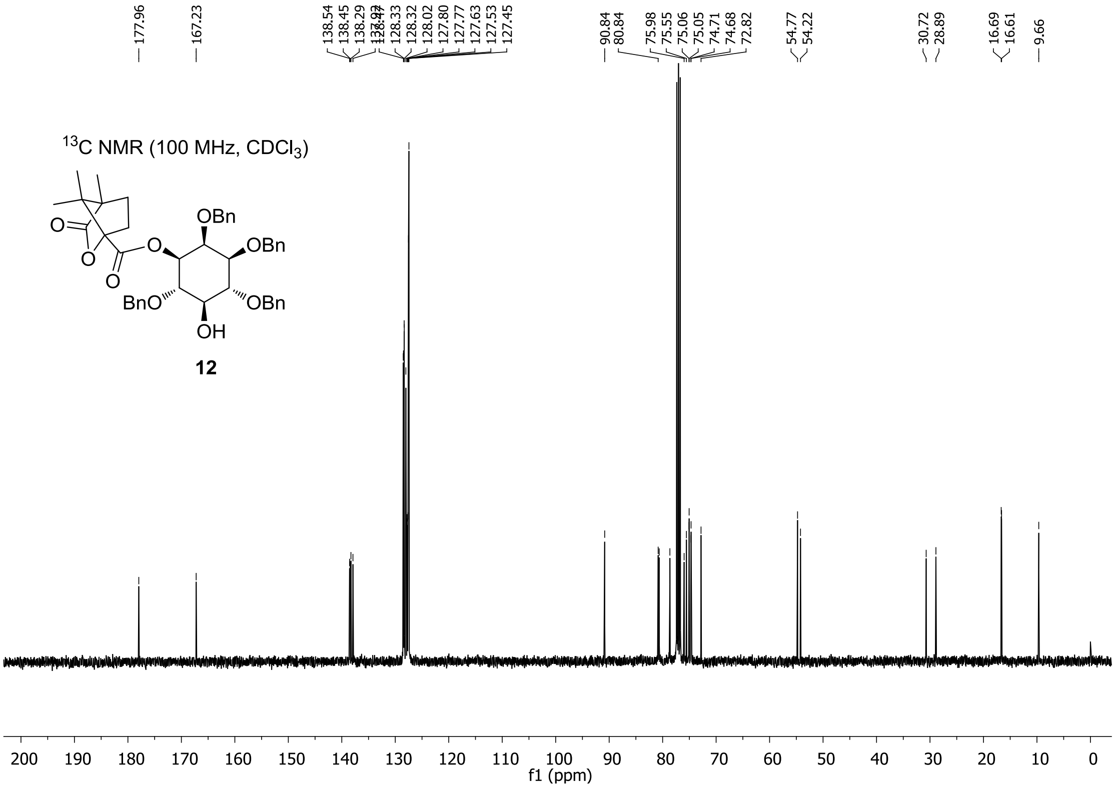

13C NMR (100 MHz, CDCl3)
OBn
OBn
BnO
OBn
OH
12
f1 (ppm)

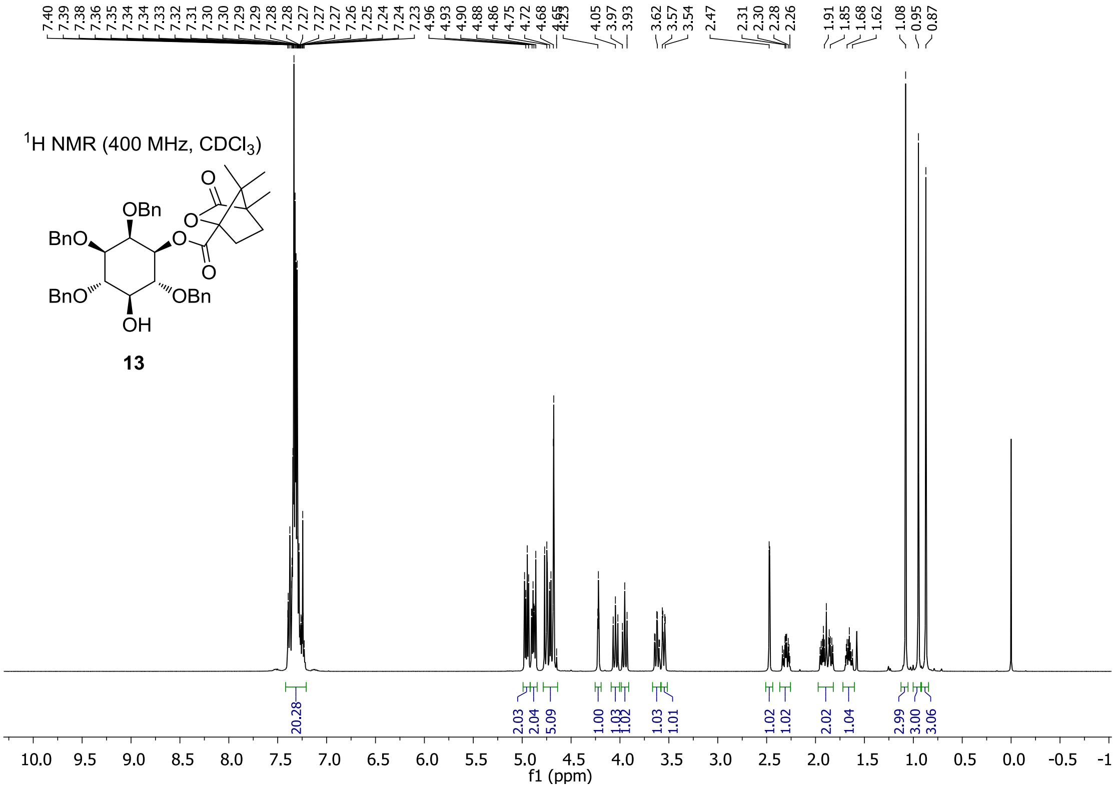
7.40 7.39 7.38 7.36 7.35 7.34 7.34 7.33 7.32 7.31 7.30 7.30 7.29 7.29 7.28 7.28 7.27 7.27 7.27 7.26 7.25 7.24 7.24 7.23 4.96 4.93 4.90 4.88 4.86 4.75 4.72 4.68 4.65 4.23 4.05 3.97 3.93 3.62 3.57 3.54 2.47 2.31 2.30 2.28 2.26 1.91 1.85 1.68 1.62 1.08 0.95 0.87
^{1}H NMR (400 MHz, CDCl$_3$)
OBn
BnO
BnO
OBn
OH
O
O
O
O
13
20.28 2.03 2.04 5.09 1.00 1.03 1.02 1.03 1.01 1.02 1.02 2.02 1.04 2.99 3.00 3.06
f1 (ppm)

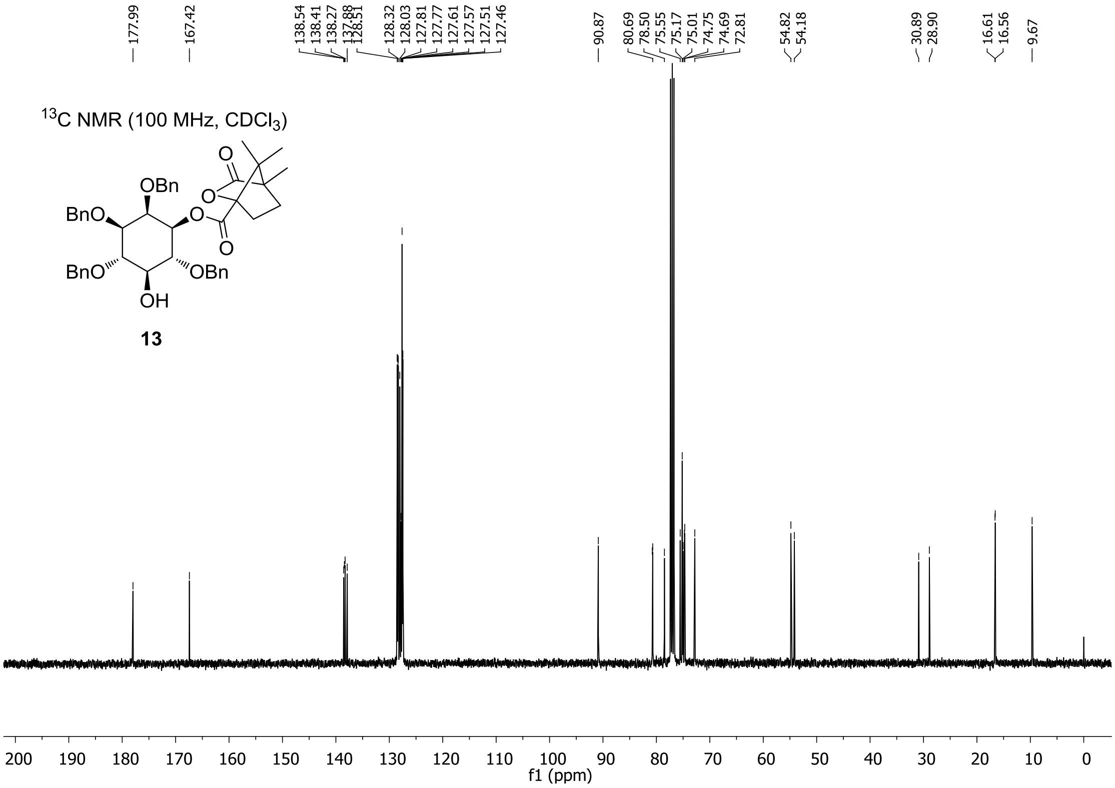
177.99 167.42 138.54 138.41 138.27 137.88 128.51 128.32 128.03 127.81 127.77 127.61 127.57 127.51 127.46 90.87 80.69 78.50 75.55 75.17 75.01 74.75 74.69 72.81 54.82 54.18 30.89 28.90 16.61 16.56 9.67
^{13}C NMR (100 MHz, CDCl$_3$)
OBn
BnO
BnO
OBn
OH
O
O
O
O
13
f1 (ppm)

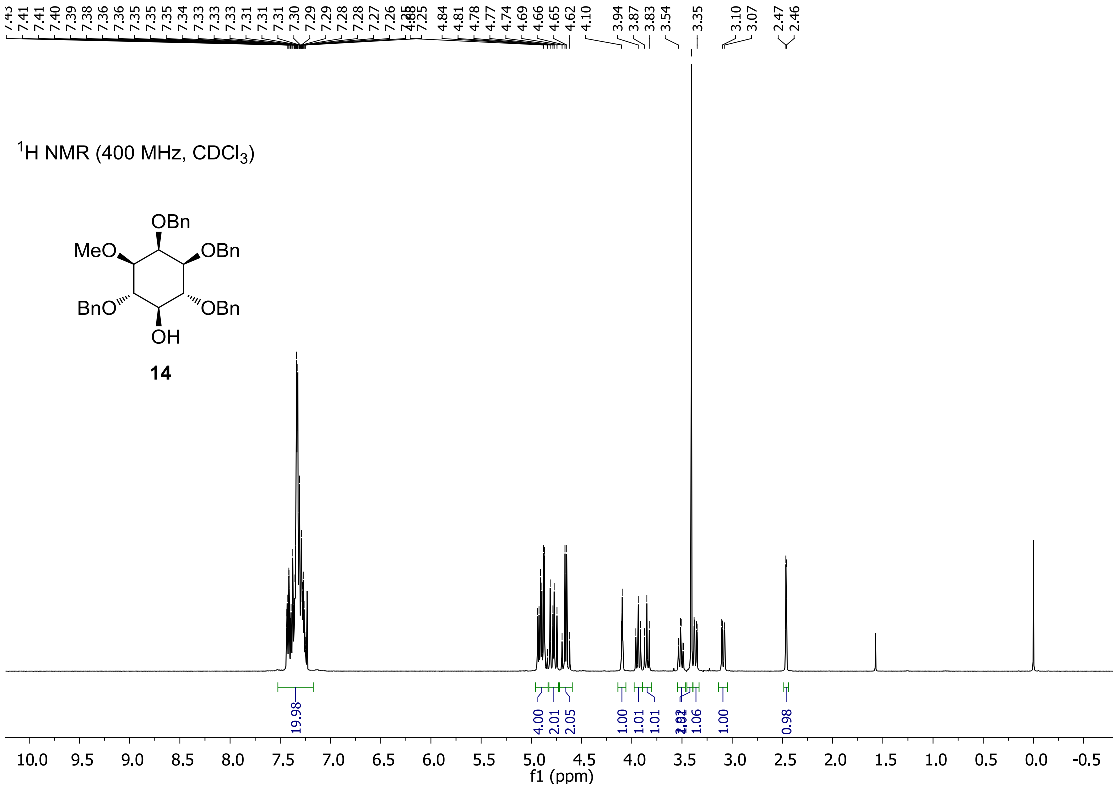

^{1}H NMR (400 MHz, $CDCl_3$)
MeO
OBn
BnO
OH
14
f1 (ppm)

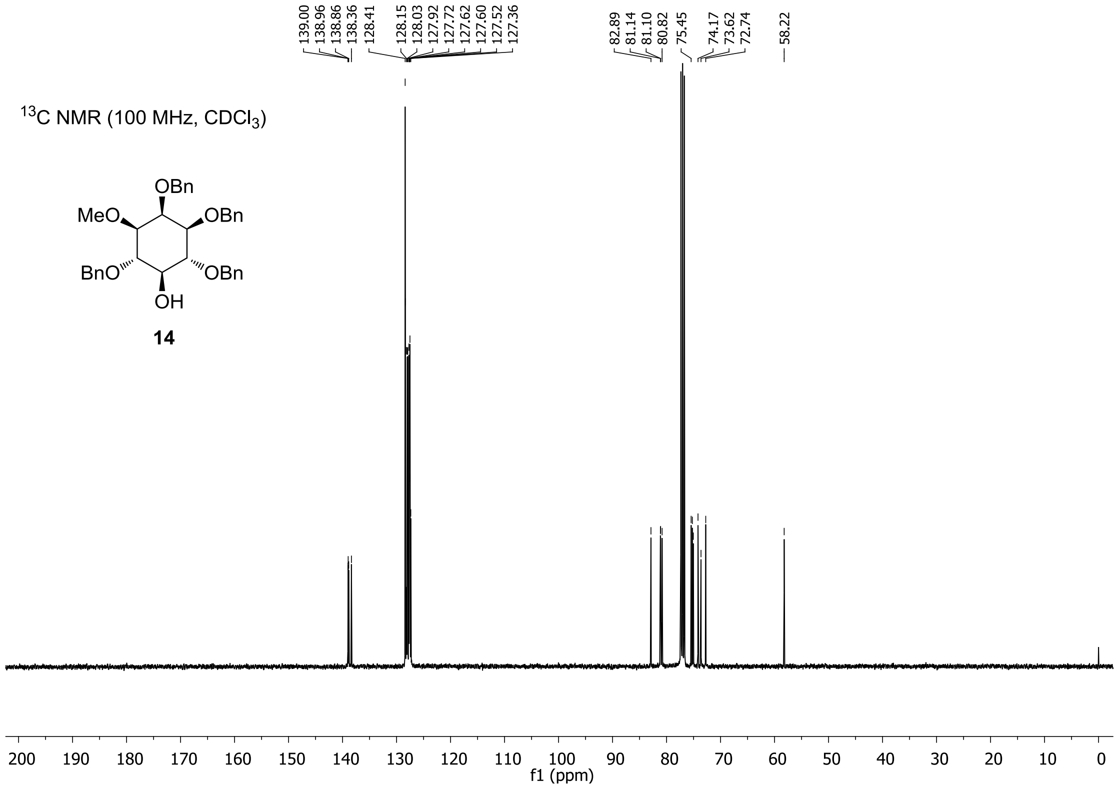

^{13}C NMR (100 MHz, $CDCl_3$)
MeO
OBn
BnO
OH
14
f1 (ppm)

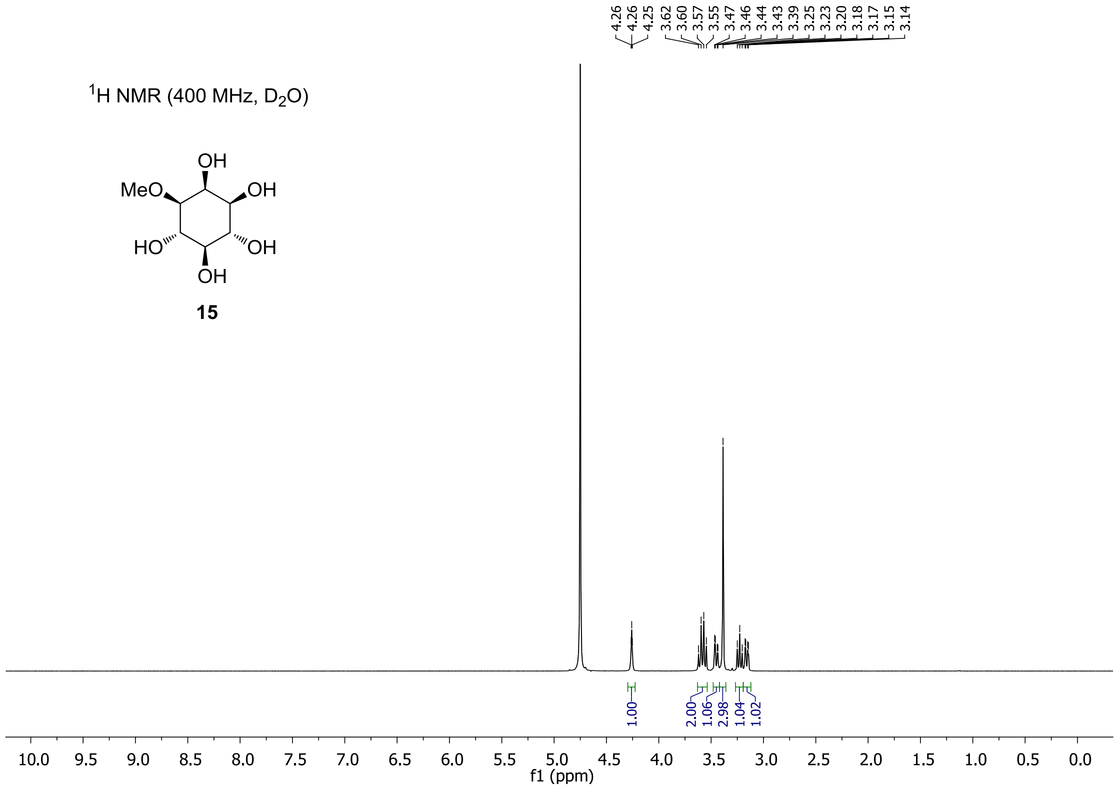

1H NMR (400 MHz, D2O)
OH
MeO
OH
HO
OH
OH
15
4.26
4.26
4.25
3.62
3.60
3.57
3.55
3.47
3.46
3.44
3.43
3.39
3.25
3.23
3.20
3.18
3.17
3.15
3.14
1.00
2.00
1.06
2.98
1.04
1.02
10.0 9.5 9.0 8.5 8.0 7.5 7.0 6.5 6.0 5.5 5.0 4.5 4.0 3.5 3.0 2.5 2.0 1.5 1.0 0.5 0.0
f1 (ppm)

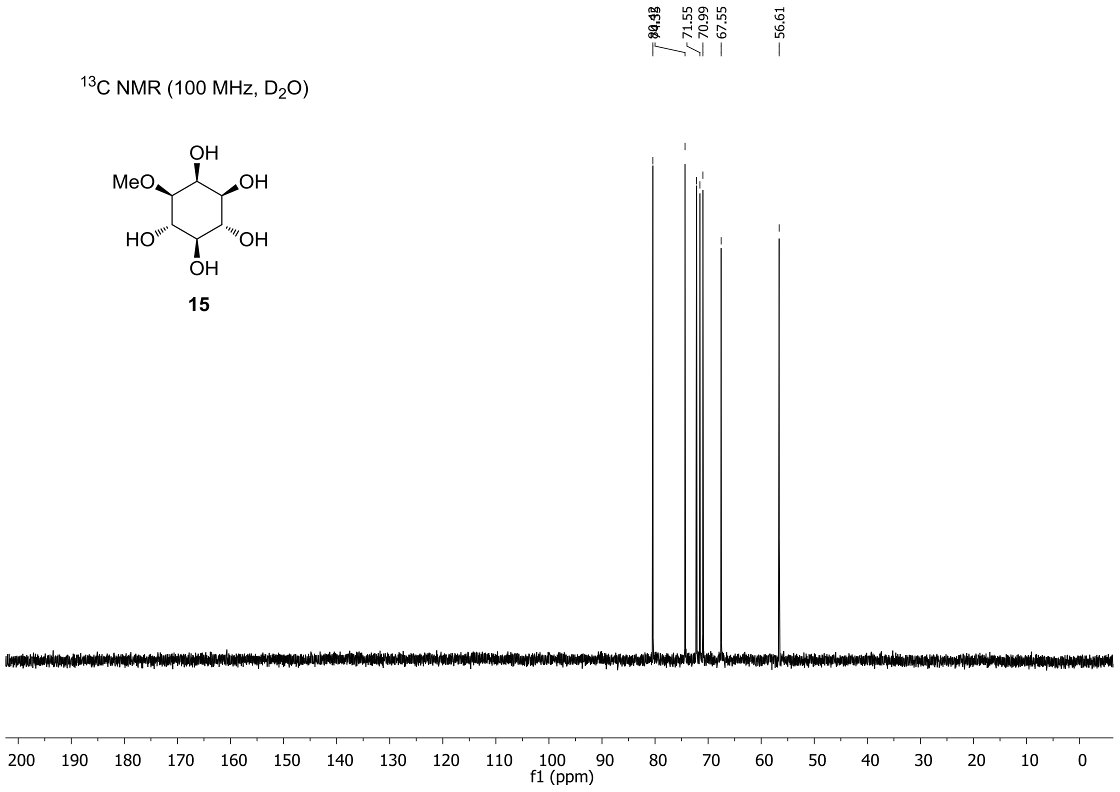

13C NMR (100 MHz, D2O)
OH
MeO
OH
HO
OH
OH
15
71.55
70.99
67.55
56.61
200 190 180 170 160 150 140 130 120 110 100 90 80 70 60 50 40 30 20 10 0
f1 (ppm)

References

1. M. Hoenig, R. J. Lee and D. C. Ferguson, *J. Biochem. Biophys. Meth*., 1989, **19**, 249–252.

2. A. M. Riley, H. Wang, J. D. Weaver, S. B. Shears and B. V. L. Potter, *Chem. Commun.*, 2012, **48**, 11292-11294.

3. J. D. Weaver, H. Wang and S. B. Shears, *Biosci. Rep.*, 2013, **33**, 229-242.

4. H. Wang, J. R. Falck, T. M. Hall and S. B. Shears, *Nat. Chem. Biol.,* 2012, **8**, 111–116.

5. C. Grison, P. Coutrot, S. Joliez and L. Balas, *Synthesis*, 1996, 731-735.

6. H. Y. Godage, A. M. Riley, T. J. Woodman, M. Thomas, M. Mahon and B. V. L. Potter, *J. Org. Chem.* 2013**, 78** 2275-2288.

7. J. M. Swarbrick, S. Cooper, G. Bultynck and P. R. J. Gaffney, *Org. Biomol. Chem.*, 2009, **7**, 1709-1715.

8. P. Westerduin, H. A. M. Willems and C. A. A. van Boeckel, *Tetrahedron Lett.*, 1990, **31**, 6915-6918.

9. A. M. Riley, H. Y. Godage, M. F. Mahon and B. V. L. Potter, *Tetrahedron-Asymmetry*, 2006, **17**, 171-174.

10. J. Gigg, R. Gigg, S. Payne and R. Conant, *J. Chem. Soc. Perkin Trans. I*, 1987, 1757-1762. See also references therein.
